# Lipidomic analysis of skeletal muscle tissues of p53 knockout mice by nUPLC-ESI-MS/MS

**DOI:** 10.1038/s41598-017-02065-9

**Published:** 2017-06-12

**Authors:** Se Mi Park, Seul Kee Byeon, Hojun Lee, Hyerim Sung, Il Yong Kim, Je Kyung Seong, Myeong Hee Moon

**Affiliations:** 1 Department of Chemistry, Yonsei University, Seoul, 03722 Korea; 2 College of Veterinary Medicine, BK21 Program for Veterinary Science and Research, Institute of Veterinary Science, Seoul National University, Seoul, 08826 Korea; 3 Korea Mouse Phenotyping Center (KMPC), Seoul, 08826 Korea

## Abstract

Tumour suppressor p53 is known to be associated with the maintenance of mitochondrial functional properties in the skeletal muscles. As deactivation or mutation of p53 can affect the synthesis of lipids, investigating the relationship between p53-related energy generation metabolism and perturbation of lipid profile is critical. In this study, 329 lipid species (among 412 identified species) in two different skeletal muscle tissues (the gastrocnemius and soleus) from p53 knockout (KO) mice were quantitatively analysed using nanoflow ultrahigh performance liquid chromatography tandem mass spectrometry (nUPLC-MS/MS). Overall, lipids from the soleus tissues were more affected by p53 KO than those from the gastrocnemius in most lipid profiles. In p53 KO, lysophosphatidylcholine (LPC), lysophosphatidylserine (LPS), phosphatidic acid (PA), sphingomyelin (SM), and triacylglycerol (TAG), including 6 TAG (44:2, 46:0, 58:5, 58:8, 58:9, and 50:0), were significantly increased (p < 0.05) by 1.4–2-fold only in the soleus tissue. Overall monohexosylceramide (MHC) levels, including those of 3 MHC species (d18:0/24:0, d18:1/22:0, and d18:1/24:0), were significantly increased (p < 0.05) by 2–4 fold, only in the gastrocnemius tissue. The results suggest that lipid profiles are significantly altered by the lack of p53 in muscle tissues.

## Introduction

The p53 tumour suppressor protein orchestrates cellular transcription programs in response to diverse stress signals, including DNA damage and oxidative stress, that ultimately lead to cell cycle arrest, apoptosis, and senescence^1, 2^. Recently, metabolic reprogramming has been recognised as an important hallmark of cancer^3^. p53 plays many roles in metabolic pathways^4, 5^. For example, the p53 tumour suppressor protein responds to metabolic stress by reprogramming cellular metabolism in tumour cells, greatly influencing tumour development and progression^4–8^. Loss-of-function mutations of the p53 tumour suppressor protein in tumour cells abrogate inhibition of cell growth under nutrition deprivation. Furthermore, highly proliferative cancer cells require more building blocks and, therefore, resort to an inefficient type of metabolism and aerobic glycolysis, in place of oxidative phosphorylation. The p53 tumour suppressor protein plays an important role in these pathways by inhibiting the glycolytic pathway and increasing oxidative phosphorylation. Thus, loss-of-function mutations of the p53 tumour suppressor protein in tumour cells induce increased glycolysis to provide tumour cells with sufficient building blocks for their proliferation. In addition, cancer cells compensate for their inefficient energy production by increasing glucose uptake^5, 9^. Given that p53 inhibits the expression of glucose transporters such as GLUT1 and GLUT4, loss-of-function mutations of the p53 tumour suppressor protein in tumour cells induce increased glucose influx into tumour cells^9^. Taken together, it seems that cancer cells reprogram metabolic pathways to favour aerobic glycolysis and glucose uptake in order to support accelerated tumour cell growth by targeting the p53 tumour suppressor protein.

While accumulating evidence demonstrates the significant roles of the p53 tumour suppressor protein in metabolic reprograming, its role in lipid metabolism remains largely unknown. Few studies showed the influence of p53 mutation on lipid profiles such as an observation in the change of unsaturated acyl chains of phospholipids (PLs) toward more saturated moieties in p53 knockout (KO) liver cells^10^, a decrease of acyl chain length of phosphatidylinositol (PI) by p53 mutation in pancreatic cancer^11^, and an increase of phosphatidylcholine (PC) levels in p53 KO colon cancer cell lines by magnetic resonance imaging (MRI) and spectroscopy (MRS)^12^. Most recently, we performed a series of lipidomic analyses of three internal organ tissues (the lung, kidney, and liver tissues) and three brain tissues (the cortex, hypothalamus, and hippocampus) derived from p53 KO mice using nanoflow liquid chromatography-electrospray ionization-tandem mass spectrometry (nLC-ESI-MS/MS) and demonstrated that p53 status altered lipid profiles in a tissue-specific manner^13, 14^. Since p53 is associated with the maintenance of mitochondrial functional properties in the skeletal muscle^15, 16^ and dysfunctional utilisation of lipids in mitochondria is associated with muscle-related diseases such as diabetes, obesity, and sarcopenia^17–20^, it is important to investigate the role of p53 in lipid metabolism of skeletal muscles.

Incorporation of nanoflow LC using a capillary column to MS analysis for lipids has shown its capabilities in handling a small amount (low femtomolar levels) of lipid species with improved resolution of separation^21, 22^, identifying a large number (>400) of lipids with molecular structures together with a high speed quantification (<20 min) using selective reaction monitoring (SRM)^13, 14, 23, 24^, and it has been successfully applied to various lipid samples from plasma, urine, and liver tissues^23–25^.

In this study, we quantify lipids from two skeletal muscle tissues of p53 KO mice using nUPLC-ESI-MS/MS to detect changes in aerobic metabolism by lipidomic perturbations in a p53-null setting: the gastrocnemius (Gas), which is involved in running by obtaining energy from both mitochondrial respiration and glycolysis, and the soleus (Sol), which is recruited in standing still by the supply of energy mainly from mitochondrial respiration. For this purpose, a non-targeted analysis of lipids, including phospholipids (PLs), glycerolipids (GLs), and sphingolipids (SLs), was performed on pooled tissue sample from wild type (WT) and p53 KO mice by nUPLC-ESI-MS/MS using an ion trap MS. Secondly, high speed targeted-quantification of 329 lipids was performed on individual animal sample for 329 lipids by nUPLC with a triple quadrupole MS using SRM. Finally, alterations in anaerobic metabolism through the change in lipid profiles of the two skeletal muscle tissues of mice lacking p53 were statistically assessed in comparison to those of WT mice.

## Results

### Lipid profiling in skeletal muscles of mice

**Figure 1:**
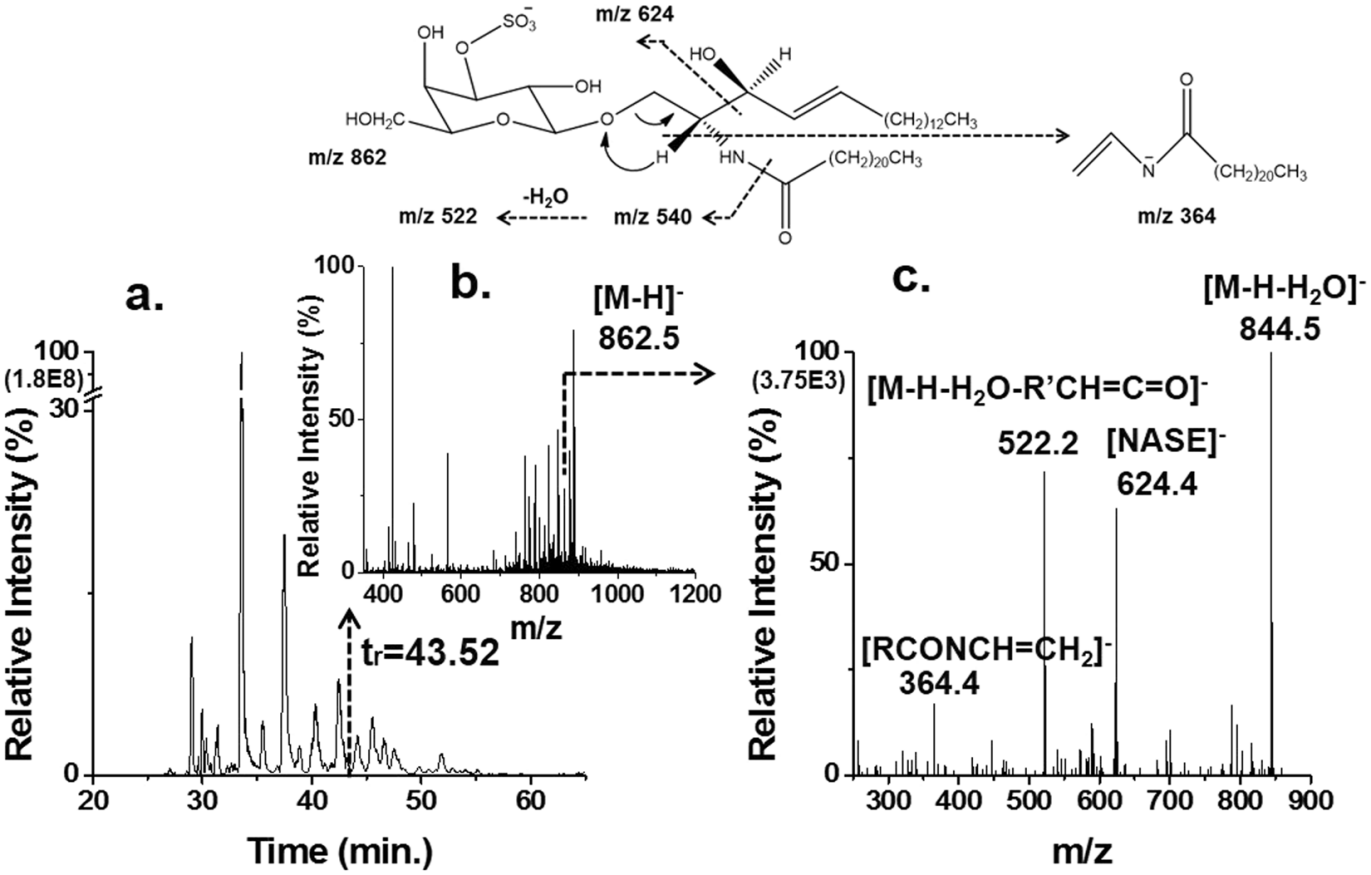
Determination of the molecular structure of a sulfatide by nLC-ESI-MS/MS. (**a**) Base peak chromatogram (BPC) of lipid extract of the gastrocnemius (Gas) tissue from wilde type (WT) mice, (**b**) a precursor scan MS spectrum at t_r_ = 38.40 min, and (**c**) the corresponding CID spectrum of m/z 862.5, [M-H]^−^, resulting in the identification of a sulfated galactosylceramide (sulfatide or ST), d18:1/22:0-ST by nLC-ESI-MS/MS.

For the targeted quantitative analysis of muscular lipids influenced by p53 KO, a comprehensive structural identification of lipid molecules from the Gas and Sol tissues of mice was accomplished by nLC-ESI-MS/MS. Identification of lipid molecular structure was based on the data dependent collision induced dissociation (CID) experiments. Figure 1 illustrates a scheme of lipid identification exemplified with a sulfated galactosylceramide (sulfatide or ST) molecule from a tissue sample by nUPLC-ESI-MS/MS: a) a base peak chromatogram (BPC) of the lipid extract sample from the Gas tissue of WT mice and b) a precursor scan MS spectrum recorded at t_r_ = 43.52 min along with c) the MS/MS spectrum obtained for the precursor ion (m/z = 862.5, [M-H]^−^). Figure 1c shows characteristic fragment ions representing the loss of water molecules ([M-H-H_2_O]^−^ at m/z 844.5) from parent ion (molecular structures shown at the top of Fig. 1), the loss of the fatty acyl chain ([M-H-H_2_O-R’CH=C=O]^−^ at m/z 522.2), N-acyl-sulphatogalactosylethylamine ion ([NASE]^−^ at m/z 624.4), and N-acyl-vinylamine ion ([RCONCH=CH_2_]^−^ at m/z 364.4), resulting in the identification of d18:1/22:0-ST. In this study, performance of lipid separation using nUPLC was demonstrated with BPC’s showing the separation of lipid standard mixtures in Supplementary Fig. S1 in positive and negative ion modes of MS. BPC’s of lipid extracts from both tissue samples are compared between WT and KO mice in Supplementary Fig. S2, resulting in the identification of 412 lipids, including 19 lipid sub-classes. Their molecular structures are listed in Supplementary Table S2. The number of identified lipids in each category is listed in Supplementary Table S1, and, among those, only 329 lipids were quantified in this study as listed in Supplementary Table S2. Since lipid quantification was based on SRM, detecting both precursor and quantifier ions, lipid classes of PC, phosphatidylethanolamine (PE), and triacylglycerol (TAG) were quantified without differentiating the isomeric combinations in acyl chains and, therefore, chain structures of these three classes are represented as the total number of carbons and double bonds in acyl chain (refer to the identified list of isomeric chain structures in Supplementary Table S3). Types of precursor and quantifier ions of each lipid class selected for quantitative analysis are listed in Supplementary Table S1, which were utilised for the targeted analysis using an SRM time-table to scan every lipid molecule during a 2-min interval (average peak width of lipid <1 min) and to cover all lipid detections within 20 min. Quantified results in Supplementary Table S2 are shown with the corrected peak area of each lipid molecule in comparison to the peak area of an internal standard (1 pmol) specific to each lipid class and the ratio (KO/WT) of the corrected peak area of each molecule along with the relative abundance (%) of each species in the corresponding class based on WT. The species with the underlined abundance values represent high abundance in each class defined as the percentage value being larger than 100/number of lipids in each class. The relative abundance values of some species such as 50:6-TG may be as small as 0.01% in Supplementary Table S2 but they were distinctly detected from the level of background noise in both Gas and Sol tissues, as shown on Supplementary Fig. S3. Under the assumption that MS intensity of lipids is not significantly affected by the length and degree of unsaturation of acyl chains, peak area ratios in Table 2 and Supplementary Table S2 can be considered as the relative values corresponding to 1 pmol of IS specific to each lipid class.

**Figure 2:**
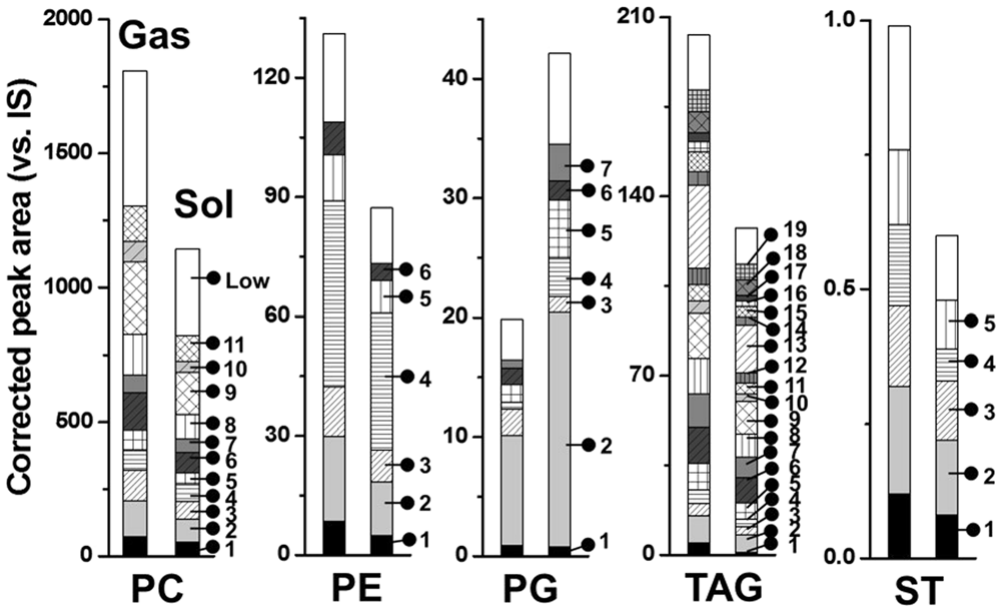
Comparison of total amounts of five lipid categories showing large differences between the Gas and Sol muscles in WT mice marked with the individual composition by nUPLC-ESI-MS/MS. Numbers representing individual molecules are relatively highly abundant species marked in Supplementary Table S2 and ‘low’ represent the summed amount of the species present in low amounts. Plots of the remaining 14 categories are presented in Supplementary Fig. S4.

**Figure 3:**
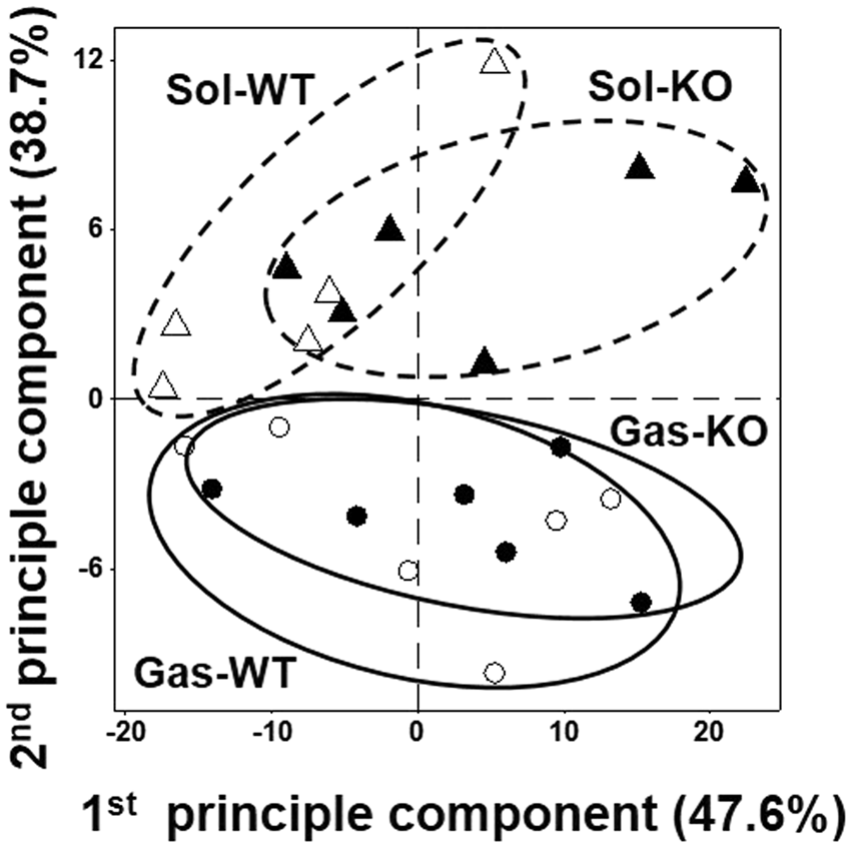
Principal component analysis (PCA) plots of the Gas (circle) and Sol (triangle) tissues showing the differences between wild type (WT, empty symbols) and p53 knockout (KO, filled symbols) mice based on quantified results of 329 lipids from each animal.

Figure 2 shows the differences in the total amounts of five lipid classes (PC, PE, phosphatidylglycerol (PG), TAG, and ST) that show a large difference (>50%) between the Gas and Sol tissues as well as the changes in compositional amounts. Molecular information of numbered species is available in Supplementary Table S2. Highly abundant species in each class along with the summed amount of remaining low abundance species marked as ‘low’ are listed. While lipid amounts of PC, PE, TAG, and ST were much lower in the Sol than those in the Gas, the amount of PG was higher in the Sol by about two-folds (Fig. 2). The other 14 classes did not exhibit noticeable differences between the Gas and Sol tissues as shown in Supplementary Fig. S4. While variations in the amounts of individual lipid molecules between the Gas and Sol appeared to be mostly similar to the differences in the total amounts between the tissues, some species were significantly different in their amounts: PG #4 (18:1/18:0-PG) and #7 (20:2/16:0-PG) were more abundant in the Sol by about four- to six-folds. Figure 3 and Supplementary Fig. S4 not only show the differences in the lipid distribution between the Gas and Sol tissues but also exhibit the relative abundance of each lipid class in muscle tissues. The amount of total PC was much larger than other classes in the following decreasing order: TAG, PE, lysophosphatidylethanolamine (LPE), sphingomyelin (SM), lysophosphatidylserine (LPS), and phosphatidylserine (PS). Table 1 lists the total amounts of lipid classes in the two tissues.

**Table 1:** Relative ratio (KO/WT) of each lipid category based on total peak area (vs. IS) by nLC-ESI-MS/MS with SRM.

Class (number)	Gastrocnemius			Soleus		
	WT	KO	KO/WT	WT	KO	KO/WT
LPC (12)	20.65 ± 5.06	16.62 ± 3.57	0.80 ± 0.26	20.05 ± 6.73	46.33 ± 10.46	2.31 ± 0.94
PC (26)	1806.74 ± 256.29	2036.32 ± 350.42	1.13 ± 0.25	1144.54 ± 127.33	1827.16 ± 233.28	1.60 ± 0.27
LPE (9)	214.37 ± 52.32	227.99 ± 59.44	1.06 ± 0.38	182.80 ± 40.63	230.65 ± 61.79	1.26 ± 0.44
PE (20)	131.15 ± 28.44	131.25 ± 25.57	1.00 ± 0.29	87.27 ± 10.84	138.77 ± 25.67	1.59 ± 0.35
pPEp (24)	19.60 ± 4.40	21.47 ± 3.82	1.10 ± 0.31	14.59 ± 2.56	18.82 ± 3.80	1.29 ± 0.34
LPG (5)	1.79 ± 0.38	2.53 ± 0.52	1.41 ± 0.42	1.39 ± 0.30	3.01 ± 0.48	2.17 ± 0.58
PG (20)	19.85 ± 3.50	15.10 ± 2.69	0.76 ± 0.19	42.15 ± 3.67	21.77 ± 3.11	0.52 ± 0.09
LPI (4)	22.88 ± 6.34	29.48 ± 8.69	1.29 ± 0.52	32.10 ± 8.74	40.20 ± 10.54	1.25 ± 0.47
PI (30)	10.77 ± 3.41	13.52 ± 3.40	1.26 ± 0.51	10.02 ± 3.00	14.64 ± 4.03	1.46 ± 0.59
LPS (5)	64.42 ± 8.60	78.13 ± 10.38	1.21 ± 0.23	48.47 ± 6.38	72.33 ± 7.88	1.49 ± 0.25
PS (30)	42.62 ± 15.63	47.87 ± 17.52	1.12 ± 0.58	41.72 ± 13.88	51.39 ± 14.62	1.23 ± 0.54
LPA (5)	0.34 ± 0.08	0.44 ± 0.15	1.32 ± 0.55	0.42 ± 0.13	0.56 ± 0.15	1.33 ± 0.54
PA (16)	12.06 ± 3.96	14.44 ± 4.18	1.20 ± 0.52	10.76 ± 3.97	17.29 ± 5.26	1.61 ± 0.77
DAG (24)	13.55 ± 2.30	15.48 ± 3.29	1.14 ± 0.31	10.86 ± 2.73	18.16 ± 4.18	1.67 ± 0.57
TAG (65)	203.12 ± 41.21	199.50 ± 32.07	0.98 ± 0.25	127.67 ± 19.68	206.48 ± 28.95	1.62 ± 0.34
ST (8)	0.99 ± 0.29	1.10 ± 0.32	1.11 ± 0.46	0.60 ± 0.15	1.49 ± 0.40	2.50 ± 0.91
SM (10)	108.25 ± 18.84	129.13 ± 17.62	1.19 ± 0.26	107.09 ± 19.98	155.72 ± 19.84	1.45 ± 0.33
Cer (9)	25.68 ± 7.39	70.82 ± 11.45	2.76 ± 0.91	30.97 ± 9.62	40.28 ± 9.56	1.30 ± 0.51
MHC (7)	17.47 ± 4.26	36.89 ± 9.01	2.11 ± 0.73	20.63 ± 6.48	21.39 ± 6.23	1.04 ± 0.44

Lipid class with underline shows significant difference between WT and KO with *p* < 0.05. The numbers in the parentheses of each lipid category represent the number of species examined in quantitative analysis.

### Effect of p53 KO on skeletal lipid profiles

**Figure 4:**
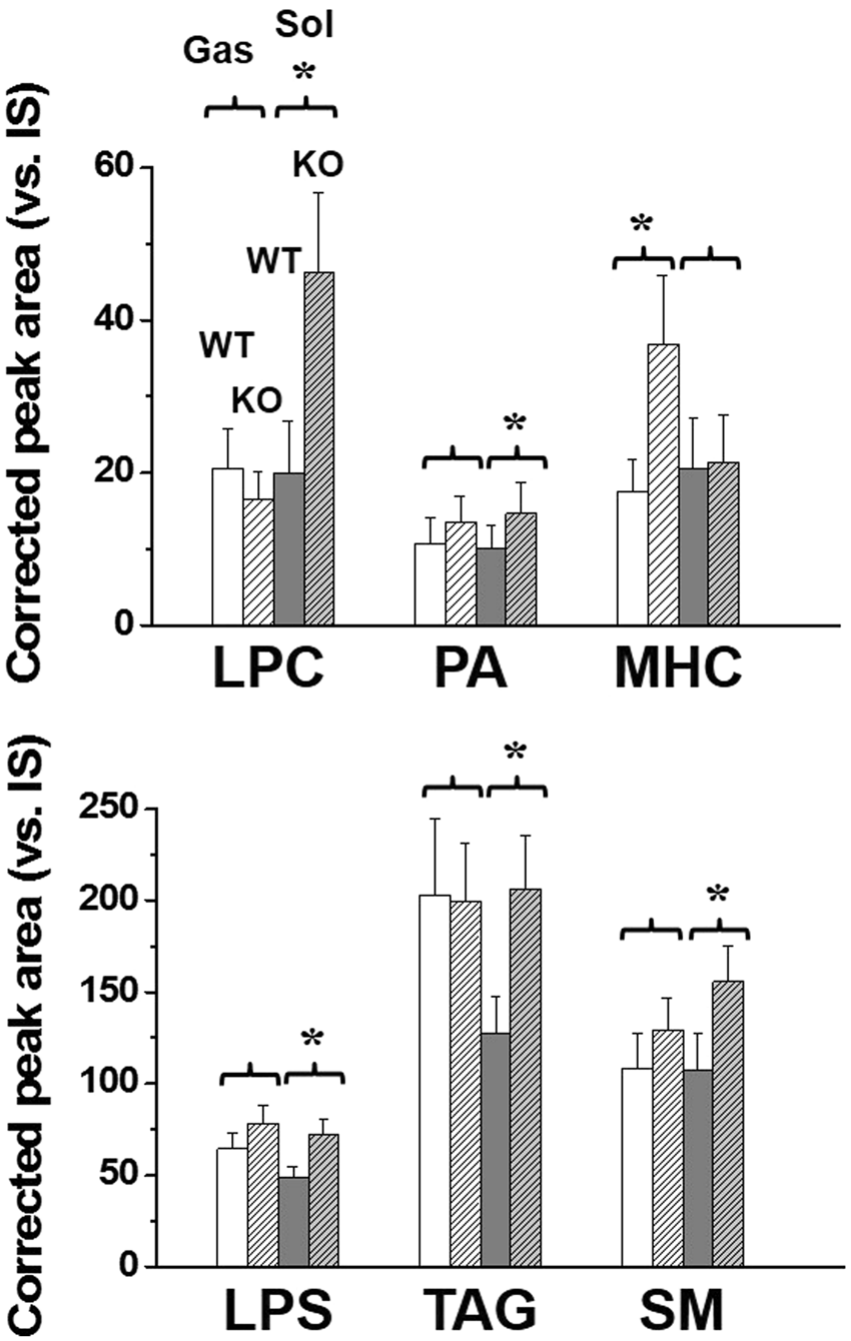
Total amounts (relative to IS of each lipid category) of LPC, LPS, PA, TAG, SM, and MHC in the Gas and Sol tissues from WT and p53 KO mice. Lipid category marked with an asterisk (*) shows a significant change (*p* < 0.05) between WT and KO mice.

When overall lipid levels were compared between WT and KO mice in each tissue, it appeared that the Sol was more influenced by the lack of p53 as shown in the principal component analysis (PCA) plots based on the quantified results of 329 lipids from six animals in each group, except for WT soleus in which only five animals were available (Fig. 3). At the lipid class level, only six classes were significantly affected (*p* < 0.05, marked with *) by p53 deficiency and are plotted in Fig. 4. Lysophosphatidylcholine (LPC), phosphatidic acid (PA), LPS, TAG, and SM increased in the Sol, but did not change in the Gas of p53 KO mice, and monohexosylceramide (MHC) only increased in the Gas tissue. However, the other 13 classes showed some degrees of change, but no statistical difference was observed as shown in Supplementary Fig. S5. The ratio (KO/WT) of the total amount of each lipid class is listed in Table 1 with the underlined classes showing significant differences in p53 KO mice. This indicates that p53 plays a pivotal role in lipid regulation in a muscle-specific manner.

**Figure 5:**
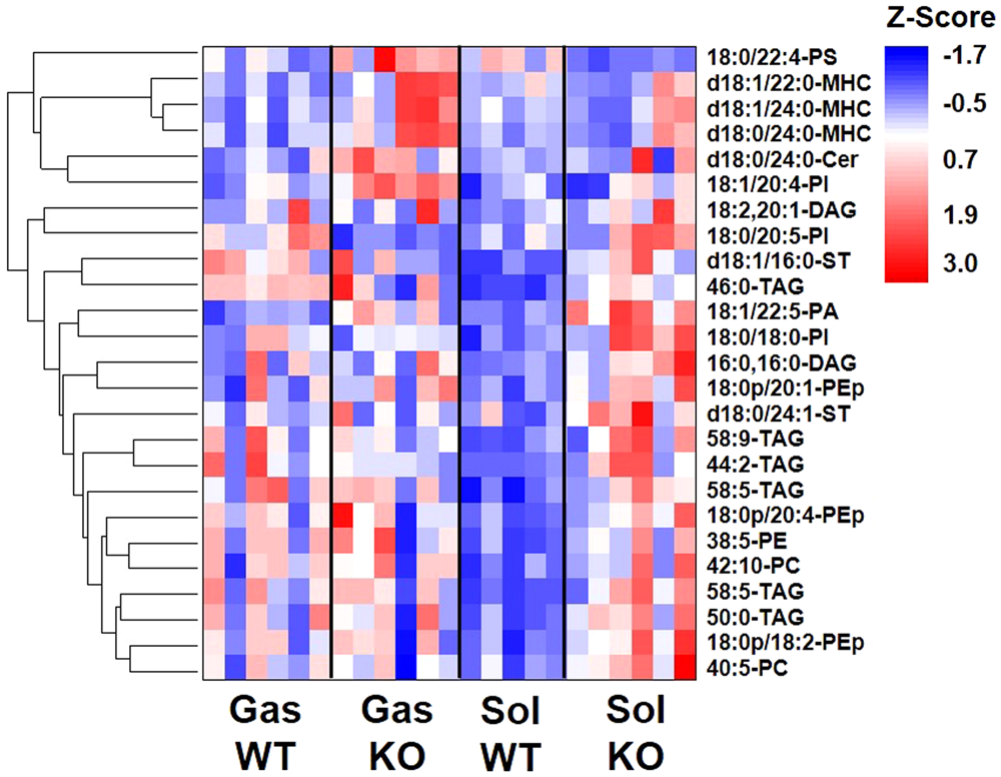
Heatmap representing lipid species showing significant changes (>2-fold, *p* < 0.05) either in the Gas or Sol tissues of WT and KO mice.

**Figure 6:**
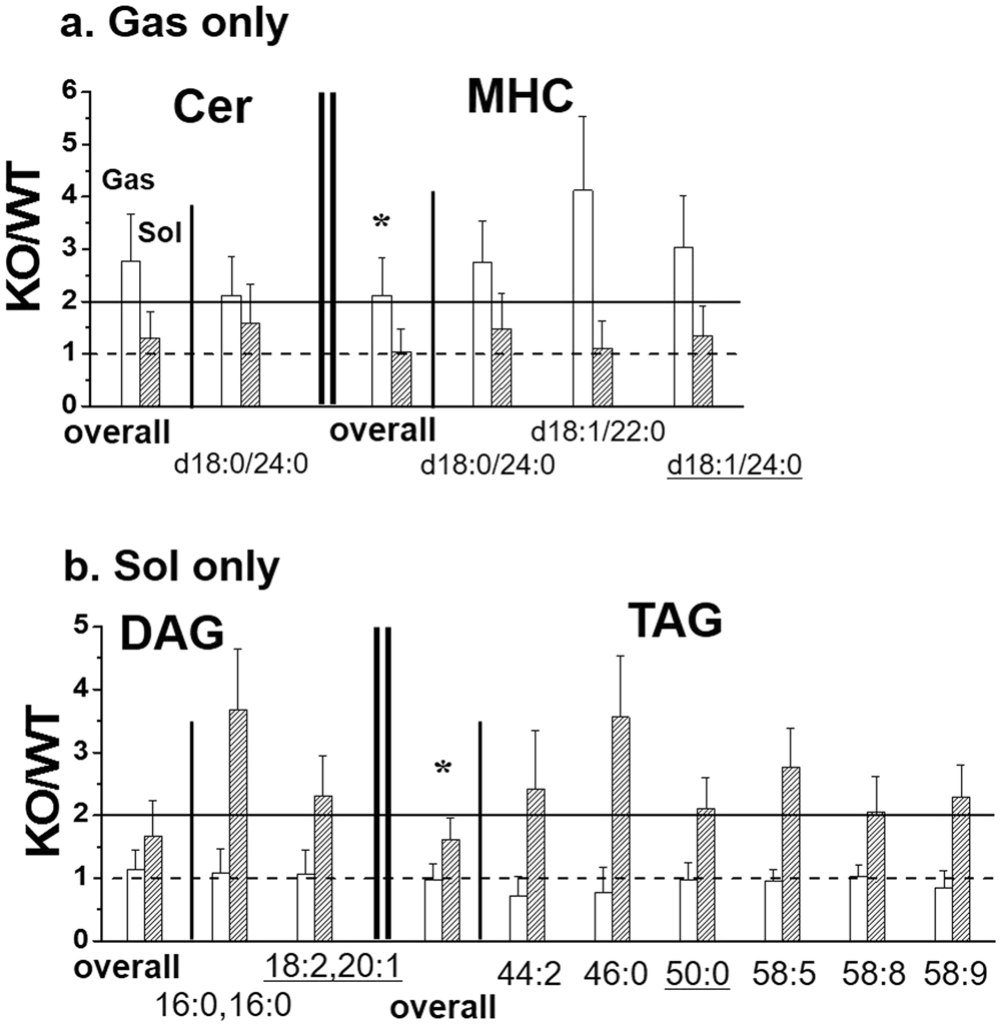
Changes (KO/WT) in a) the Cer and MHC species with a significant change (>2-fold and *p* < 0.05) in the Gas only and b) the DAG and TAG species with a significant change in the Sol only. Underlined species are the highly abundant ones.

The influence of p53 KO on molecular levels was examined for all the quantified lipid species by calculating the corrected peak area ratio (KO/WT) of each lipid species in both tissues and the calculated KO/WT values along with the relative abundance (based on WT) in each lipid class are listed in Supplementary Table S2. The species showing significant differences (*p* < 0.05) between KO and WT are marked in bold characters. Among them, lipids exhibiting more than 2-fold changes are listed in Table 2 with *p*-value. These species were plotted in a heatmap (Fig. 5), indicating that p53 KO largely influenced the Sol tissue rather than the Gas. In particular, p53 KO resulted in an increase in most species in either the Gas or Sol tissue (Table 2). However, 18:1/22:5-PA increased in both tissues, while 18:0/22:4-PS increased in the Gas, but decreased in the Sol. Moreover, the two PC (40:5 and 42:10), 38:5-PE, and the three PE plasmalogen (PEp) species (18:0p/18:2, 18:0p/20:1, and 18:0p/20:4) increased in the Sol, while they were not altered in the Gas. Figure 6 shows the comparison of the individual lipid molecules showing significant changes in a) Gas only and b) Sol only, indicating that d18:0/24:0-ceramide (Cer) and the three MHC species (d18:0/24:0, d18:1/22:0, and d18:1/24:0) were significantly increased in the Gas though they were not affected in the Sol. Additionally, the two diacylglycerol (DAG) (16:0,16:0 and 18:2,20:1) and the six TAG (44:2, 44:2, 46:0, 50:0, 58:5, 58:8, and 58:9) were not altered in the Gas, but highly elevated in the Sol. When comparing the lipid species significantly altered in internal organs (the liver, kidney, and lung) in the p53 KO mice^13^, only five species listed in Table 2 (marked with *) increased in the internal organs: 18:0/18:0-PI, 18:0/20:5-PI, 44:2-TAG, and 50:0-TAG. While Cer is known to induce apoptosis, formation of hexosylceramide like MHC from Cer allows cells to escape ceramide-induced apoptosis^26^. Although the overall Cer level was increased about 2.7 times (Fig. 6a), the relative abundance of d18:0/24:0-Cer was rather low (3.60% in Cer, Supplementary Table S2) and not significant (*p* = 0.24). However, overall MHC levels, including the high abundance d18:1/24:0-MHC (30.51% in MHC), were significantly increased in the Gas, which may indicate that the intrinsic apoptotic pathway in the Gas tissue is disturbed by the lack of p53.

**Table 2:** Peak area ratio (KO/WT) of individual lipid species **s**howing significant changes (>2-fold, *p* < 0.05, bold) either in gastrocnemius (Gas) or soleus (Sol) tissue samples along with *p* value.

Category	Molecular species	m/z	Gas			Sol		
			KO/WT	% (WT)	*p*	KO/WT	% (WT)	*p*
PC	40:5	836.5	0.94 ± 0.17	2.87	0.94	**2.13 ± 0.44**	3.21	0.02
	42:10	854.5	1.03 ± 0.30	0.60	0.81	**2.28 ± 0.56**	0.43	0.03
PE	38:5	766.6	1.02 ± 0.24	3.87	1.00	**2.08 ± 0.30**	2.64	0.04
PEp	18:0p/18:2	728.5	1.04 ± 0.30	1.12	0.48	**2.27 ± 0.34**	0.91	0.01
	18:0p/20:1	758.5	1.35 ± 0.22	0.12	0.31	**2.13 ± 0.65**	0.11	0.04
	18:0p/20:4	752.6	1.12 ± 0.33	3.01	1.00	**2.05 ± 0.32**	2.20	0.02
PI	18:0/18:0*	865.5	0.74 ± 0.34	0.71	0.93	**2.94 ± 1.23**	0.41	0.02
	18:0/20:5**	883.5	**0.48 ± 0.16**	1.02	0.01	1.62 ± 0.63	0.73	0.13
	18:1/20:4	883.5	**2.09 ± 1.01**	5.06	0.02	1.29 ± 0.69	4.51	0.43
PS	18:0/22:4	838.6	**2.05 ± 1.18**	0.21	0.03	**0.47 ± 0.23**	0.15	0.00
pPA	18:1/22:5	747.7	**2.05 ± 0.87**	13.98	0.04	**3.02 ± 1.74**	17.39	0.02
DAG	16:0, 16:0	586.5	1.08 ± 0.38	1.95	0.94	**3.68 ± 0.97**	1.12	0.02
	18:2, 20:1	664.6	1.06 ± 0.39	6.26	0.82	**2.31 ± 0.63**	4.37	0.04
TAG	44:2***	764.7	0.71 ± 0.31	0.11	0.49	**2.41 ± 0.94**	0.08	0.01
	46:0***^,†^	796.8	0.78 ± 0.39	2.31	0.81	**3.56 ± 0.98**	0.74	0.02
	50:0***	852.8	0.98 ± 0.26	2.57	1.00	**2.10 ± 0.50**	2.30	0.01
	58:5	954.8	0.95 ± 0.19	0.01	0.94	**2.77 ± 0.61**	0.01	0.01
	58:8	948.6	1.02 ± 0.20	0.09	1.00	**2.06 ± 0.56**	0.08	0.02
	58:9	946.6	0.85 ± 0.27	0.21	0.59	**2.29 ± 0.51**	0.17	0.04
ST	d18:0/24:1	890.7	1.26 ± 0.62	4.78	1.00	**2.71 ± 0.99**	6.54	0.04
	d18:1/16:0	778.6	0.74 ± 0.34	20.55	0.31	**5.01 ± 1.71**	6.00	0.02
Cer	d18:0/24:0	652.5	**2.11 ± 0.74**	3.60	0.03	1.58 ± 0.74	2.83	0.93
MHC	d18:0/24:0	814.7	**2.74 ± 0.79**	1.05	0.04	1.47 ± 0.68	0.87	1.00
	d18:1/22:0	784.7	**4.12 ± 1.41**	9.68	0.04	1.11 ± 0.51	14.92	0.61
	d18:1/24:0	812.7	**3.04 ± 0.98**	30.51	0.04	1.35 ± 0.57	34.07	1.00

Relative abundance is expressed with peak area percentage (%) of each species within the corresponding lipid class and the underlined species are the relatively high abundance species (>100%/# of identified species within the category). Marked with extra symbols are common species reported with the significant change (p < 0.05) in kidney (*), lung (**), and liver (***) of p53 KO mice model, and skeletal muscle tissue (^†^) of diabetic rat model. Under the assumption that MS intensity of lipids is not significantly affected by the. length and degree of unsaturation of acyl chains, peak area ratios can be considered as the relative values corresponding to 1 pmol of IS.

## Discussion

The present study demonstrated that lipid levels were altered in skeletal muscles of p53 KO mice, with a larger degree of changes observed in the Sol than in the Gas. Because p53 serves as an important mediator for mitochondrial functional properties^15, 16^ and a critical mitochondrial enzyme such as cytochrome c oxidase present a 20-fold higher expression in the Sol than in the Gas^27^, lipid profiles in mitochondria-rich Sol tissue can be more influenced by p53 KO than those in the Gas, which consists of mixed fibres that rely both on mitochondrial respiration and glycolysis to generate energy^28^. As the change in the Sol was more significant than that in Gas, it can be deduced that change in lipids of the Sol is associated with the alteration of mitochondrial function in the skeletal muscle tissue, as Sol is mitochondria-rich while Gas is not. This explains LPC can be an important lipid intermediate to mitochondrial dysfunction and insulin resistance since LPC was significantly increased in Sol tissue of a diabetic rat model compared to those of a lean control by 1.73- to 3.9-folds^29^. PA serves as a major precursor of cardiolipin^30^, unique in the inner wall of the mitochondrial membrane. Thus, it can be thought that the conversion of PA to cardiolipin was interrupted by the lack of p53, leading to an accumulation of PA in the Sol. Overall, the elevated phospholipids derived from LPC, LPS, and PA in our study are related with membrane synthesis in proliferating cells, which may be indicative of cancer development^31^. However, further studies are needed to assess the relationship between p53 KO and mitochondria after a proper isolation of mitochondria or analysis from additional tissue types, such as fast-twitch extensor digitorium longus (EDL), is achieved in future.

In this study, TAG was significantly increased in the Sol, while not altered in the Gas of p53 KO mice. TAG is stored in the skeletal muscle tissue as a form of lipid droplet and the utilisation of intramyocellular TAG is limited due to the mitochondrial dysfunction, leading to insulin resistance and further accumulation of TAG under various pathological muscle conditions such as diabetes and sarcopenia^32, 33^. In this experiment, six TAG species (44:2, 46:0, 50:0, 58:5, 58:8, and 58:9) were significantly increased in the Sol, but not in the Gas of p53 KO mice (Table 2 and Fig. 6), while TAG species of different acyl chains, except the commonly increased 46:0-TAG (Table 2), were significantly increased in the Sol of diabetic rat models in an earlier study^29^. This can be inferred that accumulation of a certain type of intramyocellular TAG selectively occurs by changes in p53-mediated mitochondrial function and their changes are specific to muscle types.

The present study suggests that p53 acts as an important player in the regulation of lipids in oxidative fibres rather than in glycolytic fibers, indicating that p53 has the capacity to regulate lipid profiles possibly through changes in mitochondrial properties in the skeletal muscle, based on significant changes observed from p53 KO Sol tissue. The relationship between p53 and mitochondria will be investigated and confirmed more thoroughly using fast-twitch EDL, which is the opposite type of mitochondria-rich and slow-twitch Sol. The evidence described in this study opens new avenues for the development of biomarkers for clinical studies and early diagnosis, although there is limited amount of information on p53-mediated muscle-derived cancer development in the literature. Further studies are warranted to investigate causality between lipid profiles and carcinogenesis in the skeletal muscle, and systematic examinations of lipid profiles in combination with exercise and dietary treatments will be useful to evaluate p53-mediated regulation of lipid profiles.

## Methods

### Materials and reagents

Forty four lipid standards were utilised for optimisation of nUPLC-ESI-MS/MS conditions: 12:0-LPC, 18:1-LPC, 12:0/12:0-PC, 13:0/13:0-PC, 14:0/16:0-PC, 16:0/16:0-PC, 18:1/18:0-PC, 20:0/20:0-PC, 14:0-LPE, 18:0-LPE, 12:0/12:0-PE, 14:0/14:0-PE, 18:0/22:6-PE, 12:0-lysophosphatidylglycerol (LPG), 14:0-LPG, 18:0-LPG, 12:0/12:0-PG, 15:0/15:0-PG, 16:0/16:0-PG, 18:0/18:0-PG, 16:0/18:2-PI, 12:0/12:0-PS, 14:0/14:0-PS, 16:0/16:0-PS, 12:0-lysophosphatidicacid (LPA), 18:0-LPA, 14:0/14:0-PA, d18:0/12:0-SM, d18:1/16:0-SM, d18:1/18:0-SM, d18:1/12:0-Cer, d18:1/14:0-Cer, d18:1/22:0-Cer, d18:1/12:0-glucosylceramide (GluCer), d18:1/18:0-GluCer, d18:1/16:0-lactosylceramide (LacCer), d18:1/16:0-galactosylceramide (GalCer), (18:1)_4_-cardiolipin (CL), 16:0/18:1-DAG, 18:1/18:1-DAG, 54:1-TAG, 18:0p/22:6- PC plasmalogen (PCp), 18:0p/22:6-PEp, and d18:1/24:0-ST. The list of 19 internal standards (ISs) is provided in Supplementary Table S4. All lipid standards were purchased from Avanti Polar Lipids, Inc. (Alabaster, AL, USA) and Matreya, LLC. (Pleasant Gap, PA, USA).

NH_4_HCO_2_ and NH_4_OH were purchased from Sigma-Aldrich Co. LLC. (St. Louis, MO, USA). Methyl-tert-butyl ether (MTBE) and HPLC grade solvents (H_2_O, CH_3_CN, CH_3_OH, and isopropanol (IPA)) were purchased from Avantor Performance Materials, Inc (Center Valley, PA, USA). Fused silica tubes of 75 and 100 *μ*m I.D. (360 *μ*m O.D.) for capillary column were purchased from Polymicro Technology, LLC (Phoenix, AZ, USA). Column packing materials were Watchers® ODS-P C-18 particles (3 *μ*m and 100 Å) purchased from Isu Industry Corp. (Seoul, Korea) for non-targeted lipid identification experiments and 1.7 *μ*m ethylene bridged hybrid (BEH) particles unpacked from XBridge® BEH C18 column (1.7 *μ*m, 2.1 mm × 100 mm) purchased from Waters (Milford, MA, USA) for targeted quantitation.

### Animals

Mice employed in this study were five-month-old male p53 KO mice from the Jackson Laboratory (Bar Harbor, ME, USA) and C57BL6/N mice (as wild type or control), maintained in the animal facility at Seoul National University. Information regarding gender, food intake, and weight of mice used in this study is listed in Table S5. p53 KO mice can generate tumors as early as 3 months of age and we selectively used five-month-old mice in this study, which is the age of mice where other studies on p53 KO have used^34^, in order for them to be fully prone to occurrence of tumor. This study specifically provides lipidomic analysis of p53 KO mice at 5 month of age with possible occurrence of tumors. Animals were fed with a normal diet, NIH-31 from Zeigler Bros, Inc. (Gardners, PA, USA) *ad libitum* with tap water and euthanized with CO_2_. All animal experiments in this study followed the ‘Guide for Animal Experiments’ edited by Korean Academy of Medical Sciences and were approved by the Institutional Animal Care and Use Committee (IACUC) of Seoul National University (Permit Number: SNU-160602-15).

### Lipid extraction from skeletal tissues

Twenty-four tissue samples (the gastrocnemius and soleus tissues from WT mice (n = 6) and p53 KO (n = 6) mice) from the Korea Mouse Phenotyping Center (KMPC) were examined in this study. Each dried tissue sample was crushed into powder to obtain homogeneous mixtures. For qualitative analysis of lipids, four pooled tissue samples were prepared from each group (Gas-WT, Gas-KO, Sol-WT, and Sol-KO) by taking an equal aliquot from each animal. From each pooled sample, 2 mg were used for lipid extraction. For targeted quantitation, 2 mg of each individual animal sample were used for extraction. Due to the shortage of powder samples from the Sol-WT, only 5 individual samples of Sol-WT were analysed.

Lipid extraction was based on the Folch method, but modified with MTBE/CH_3_OH^35^, which yielded higher extraction efficiencies in most lipid categories. Each 2 mg of tissue sample was dissolved in 300 *μ*L of CH_3_OH and placed in an ice bath for 10 min. Then 1 mL of MTBE was added to the mixture, vortexed for 1 h, followed by the addition of 250 *μ*L of MS-grade H_2_O, and vortexed for 10 min in room temperature. The sample mixture was centrifuged at 1000 × *g* for 10 min and the resulting upper organic layer was transferred to a new tube. The remaining aqueous layer was mixed with 300 *μ*L of CH_3_OH, followed by 2 min of sonication and 10 min of centrifugation at 1000 × *g* in sequence. The resulting organic layer was mixed with the previously collected organic layer. The tube was wrapped with 0.45 µm MillWrap PTFE membrane from Millipore (Bedford, MA, USA) to avoid lipid evaporation during a half day of freeze-drying process. Dried lipids were dissolved in CHCl_3_:CH_3_OH (3:7, v/v) and stored at −30 °C. For nLC-ESI-MS/MS analysis, the frozen lipid sample was thawed and the concentrations of all tissue samples were adjusted to 5 μg/μL in CH_3_OH:H_2_O (9:1, v/v). The volume added to each frozen sample to adjust the concentration was utilised as the volume factor to calculate the final amount (peak area) of each lipid.

### Nanoflow LC-ESI-MS/MS of lipids

Lipid analysis of tissue samples was carried out by two steps: non-targeted lipid identification using nLC-ESI-MS/MS that consists of a model 1200 capillary LC pump system, including an autosampler from Agilent Technologies (Santa Clara, CA, USA) coupled with a model LTQ Velos ion trap mass spectrometer from Thermo Scientific (San Jose, CA, USA), first, and high speed targeted quantification using nUPLC-ESI-MS/MS assembled with a model nanoACQUITY UPLC from Waters coupled with a TSQ Vantage triple stage quadrupole MS from Thermo Scientific. LC columns utilised were homemade pulled-tip capillary columns prepared in the laboratory. Details on manufacturing columns and nUPLC-ESI-MS/MS are provided in the Supplementary Information.

For non-targeted lipid profiling, lipid samples (10 μg each) were loaded to the analytical column with mobile phase A at 600 nL/min for 10 min with the split flow valve off. Gradient elution began with the pump flow rate set to 8 μL/min to reduce dwell time with the split valve on so that the column outflow rate was adjusted at 300 nL/min. The mobile phase B was ramped to 60% over 1 min, increased to 90% for 5 min, 100% over 25 min, and maintained at 100% for 25 min. It was then resumed to 100% A in 1 min and re-equilibrated for 17 min. The ESI voltage was 3.0 kV for both ion modes. Mass range for lipids was 300–1000 amu for positive ion mode and 350–1000 amu for negative ion mode. For MS/MS analysis, 40% collision energy was applied for the data dependent CID analysis. Molecular structures of lipids were determined by LiPilot, a computer software program to determine lipid molecular structures from CID spectra, developed in our laboratory^36^.

For targeted quantitation, a faster gradient elution condition (25 min for each run) was utilised with nUPLC. Sample loading included 10 μg of lipid extracts from each tissue added to a mixture of 19 ISs at 1 μL/min of 100% mobile phase A for 15 min. Gradient elution began with 50% B for 1.5 min at a pump flow rate of 11 μL/min and a column flow rate of 300 nL/min with the split valve on. The mobile phase B was ramped to 80% for 2.5 min, further to 100% for 4 min, and maintained at 100% for 12 min. It was then resumed to 0% B in 1 min and maintained for 4 min before the next run. Quantification was made with SRM by detecting a precursor ion and a quantifier ion from fragment ions in data dependent CID experiments. Ion detection was performed in positive and negative ion modes alternatively with the scan width at m/z 2, the scan time at 0.01 sec, and ESI voltage at 3 kV. Lipid categories detected in positive ion mode were LPC, PC, LPE, PE, PEp, DAG, TAG, ST, SM, Cer, and MHC and those in negative ion mode were LPG, PG, LPI, PI, LPS, PS, LPA, and PA. Voltages for CID experiments varied depending on lipid categories: 50 V for ST, 40 V for LPC, PC, LPG, PG, LPI, PI, LPS, PS, LPA, PA, and SM, 30 V for Cer and MHC, 25 V for DAG and TAG, and 20 V for LPE, PE, and PEp. Quantitation of each lipid species was performed by calculating the corrected peak area in comparison to the peak area of the internal standard corresponding to each lipid category added to the sample. Data analysis was performed with Mann-Whitney U-test and Student’s t-test using SPSS software (version 20.0, IBM Corp., Armonk, NY, USA), and principal component analysis (PCA) using Minitab 17 statistical software (http://www.minitab.co.kr).

## Electronic supplementary material

Supplementary Information
