# Supplementary material for: Lipidomic analysis of skeletal muscle tissues of p53 knockout mice by nUPLC-ESI-MS/MS
Source: Sci Rep. 2017 Jun 12;7:3302. doi: 10.1038/s41598-017-02065-9 (PMC5468235; doi:10.1038/s41598-017-02065-9)
Supplement: Supplementary file 1 — Supplementary Information [file 41598_2017_2065_MOESM1_ESM.doc]

**Supplementary Information**

Lipidomic analysis of skeletal muscle tissues of p53 knockout mice by nUPLC-ESI-MS/MS

Se Mi Park,^1^ Seul Kee Byeon,^1^ Hojun Lee,^2,3^ Hyerim Sung,^2,3^ Il Yong Kim, ^2,3^ Je Kyung Seong,^2,3*^ and Myeong Hee Moon^1*^

^1^Department of Chemistry, Yonsei University, Seoul 03722, Korea

^2^College of Veterinary Medicine, BK21 Program for Veterinary Science and Research, Institute of Veterinary Science, Seoul National University, Seoul 08826, Korea

^3^Korea Mouse Phenotyping Center (KMPC), Seoul 08826, Korea

Table of Contents

nUPLC-ESI-MS/MS S2

Figure S1 S3

Figure S2 S4

Figure S3 S5

Figure S4 S6

Figure S5 S7

Table S1 S8

Table S2 S9

Table S3 S17

Table S4 S20

Table S5 S21

**nUPLC-ESI-MS/MS**

Analytical columns were packed with the following beads under N_2_ gas (1000 psi): Watchers® ODS-P C18 resins (3 *µ*m-100 Å) in a 7 cm long capillary (75 *µ*m I.D. and 360 *µ*m O.D.) for non-targeted analysis and 1.7 μm ethylene-bridged hybrid (BEH) particles (130 Å) in a 7 cm (0.5 cm of column tip was filled with the 3 *µ*m Watchers® ODS-P C18 resins and the remaining 6.5 cm with 1.7 μm particles) long capillary (100 *µ*m I.D. and 360 *µ*m O.D.) for targeted quantitation. The column was connected with a pump using a capillary tube via a PEEK microcross from IDEX (Oak Harbor, WA, USA) where the other two ports were connected to a Pt wire for the supply of ESI voltage and the split flow valve to split the flow along with a pressure tube (20 *µ*m I.D. capillary). Mobile phase solutions for binary gradient elution used for both analysis steps were H_2_O:ACN (9:1, v/v) for A and IPA:CH_3_OH:ACN (6:2:2, v/v/v) for B, which were added with a mixed ionization modifier (0.05% NH_4_OH and 5 mM NH_4_HCO_2_) that can be used in both positive and negative ion modes.


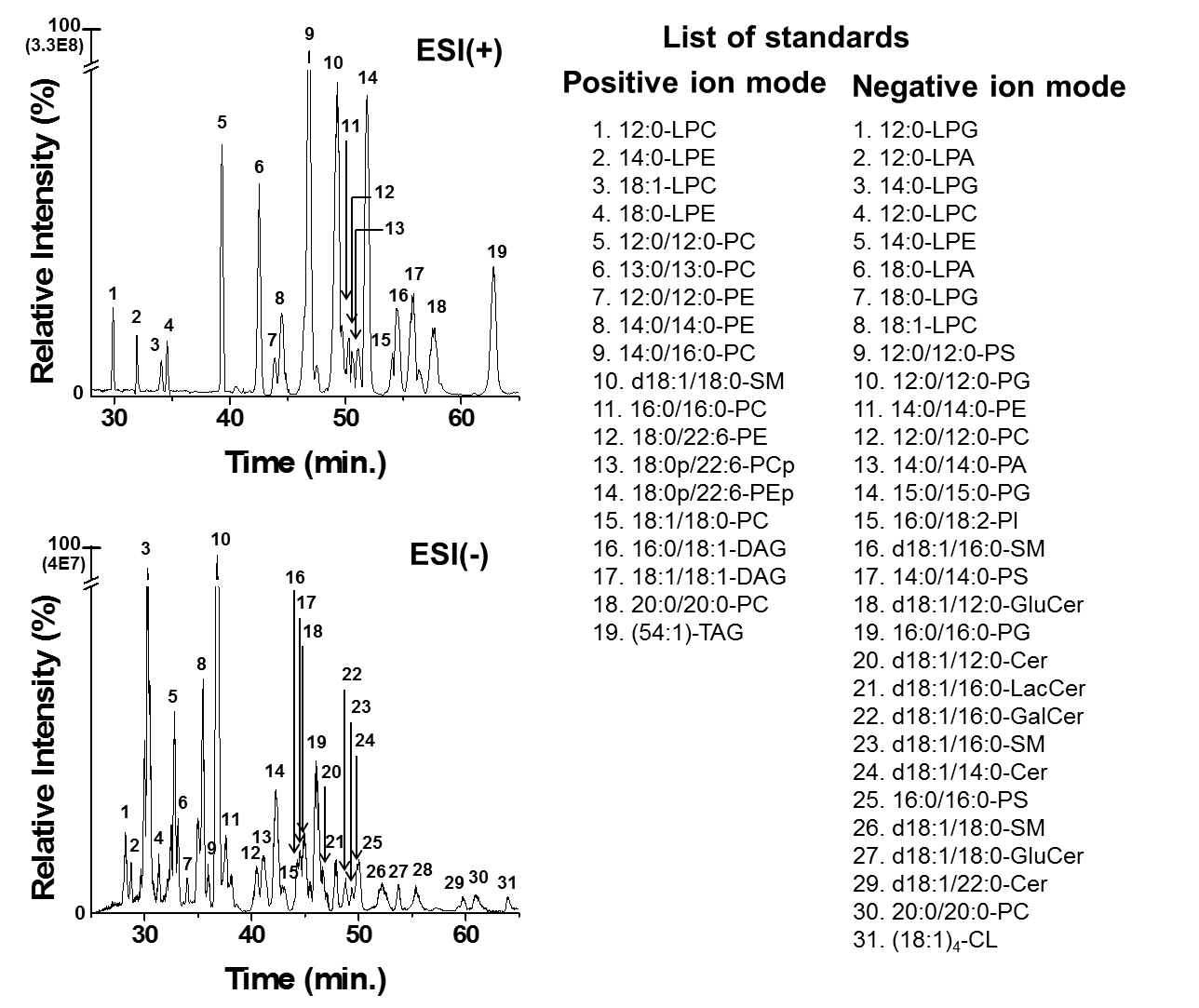


Figure S1. Base peak chromatograms (BPC’s) of 44 lipid standards in both positive and negative ion modes of nUPLC-ESI-MS/MS.


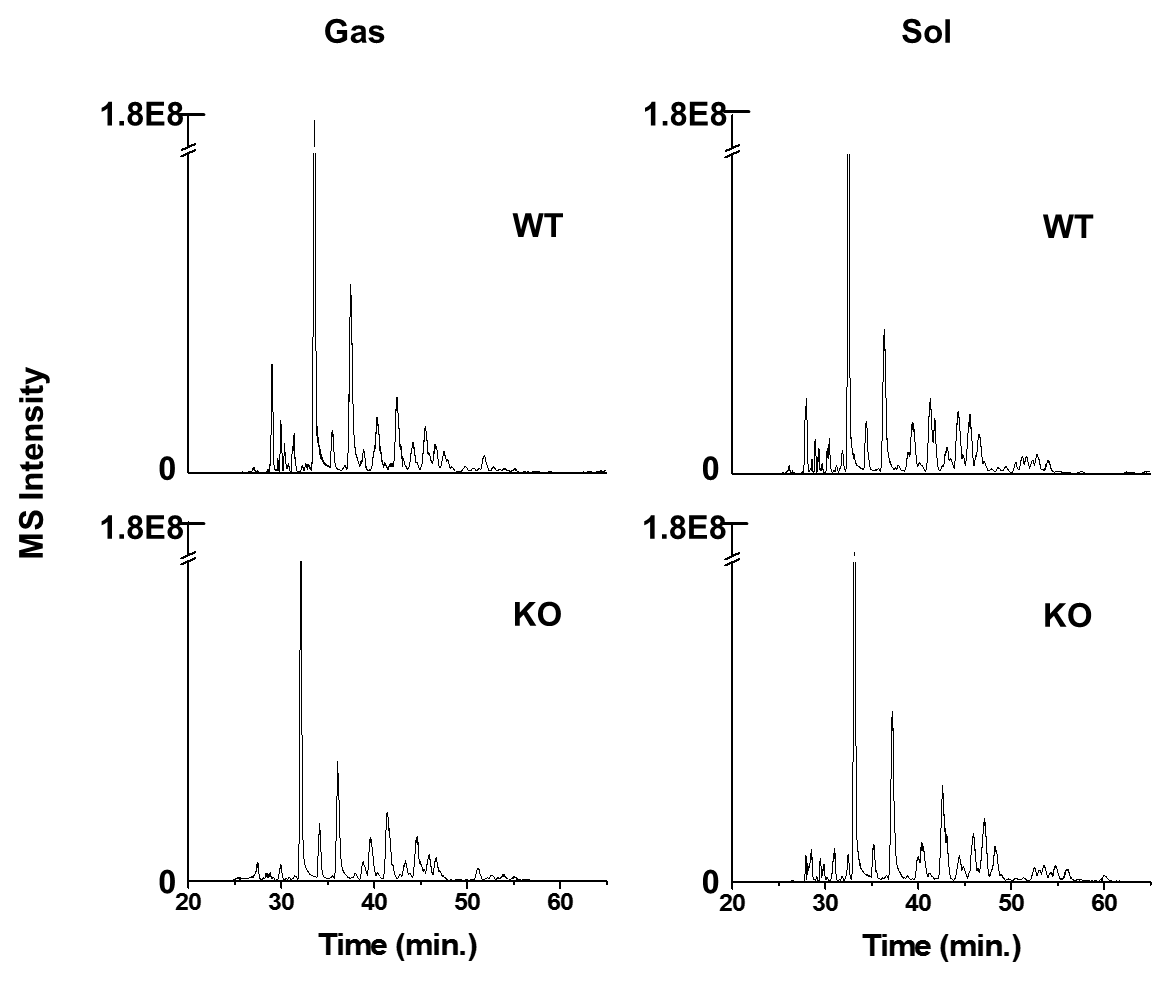


Figure S2. BPC’s of lipid extracts from the Gas and Sol tissues of mice with WT and p53 KO obtained in the negative ion mode of nUPLC-ESI-MS/MS.


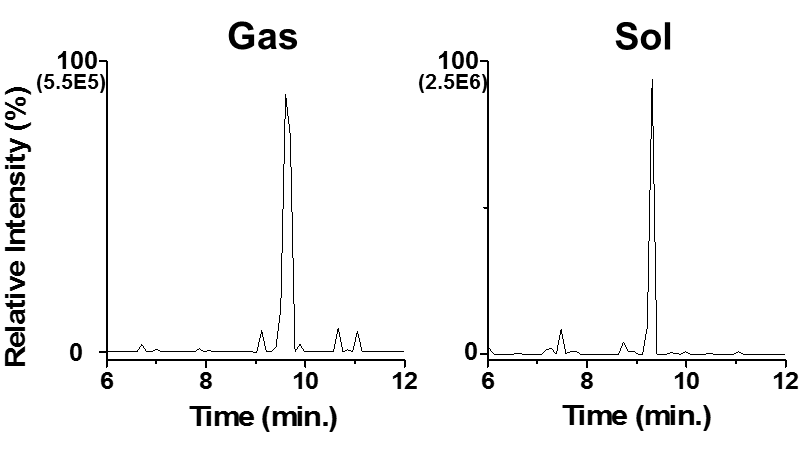


Figure S3. SRM chromatogram of low abundant species, 50:6-TG (m/z 840.7→570.4, 599.4), from Gas and Sol tissues of WT.


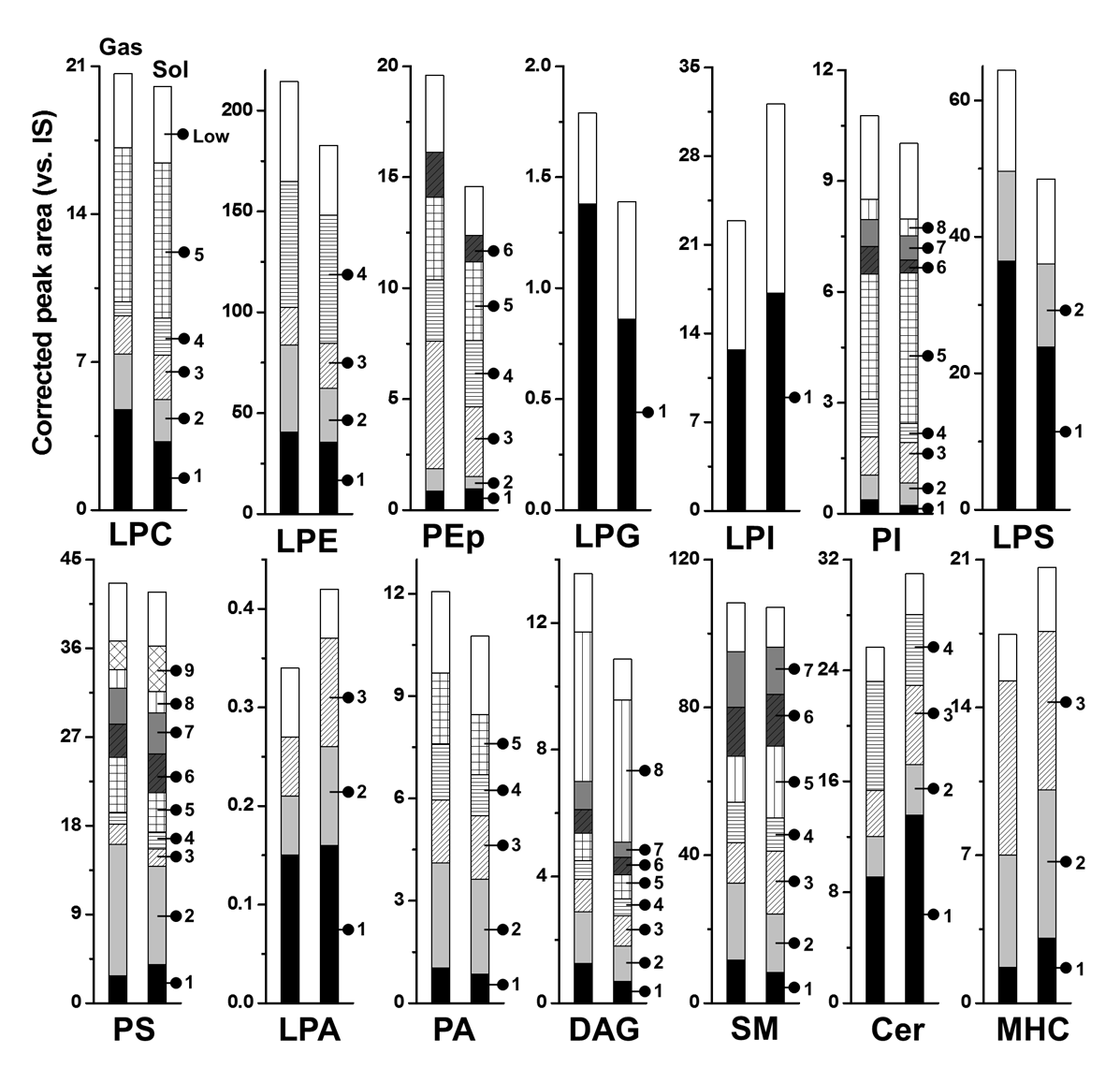


Figure S4. Comparison of total amounts of 14 lipid categories (remaining part of Figure 2) between Gas and Sol muscles of WT mice by nUPLC-ESI-MS/MS. Numbers representing individual molecules are relatively high abundance species marked in Table S4 and “low” represent the summed amount of the low abundance species.


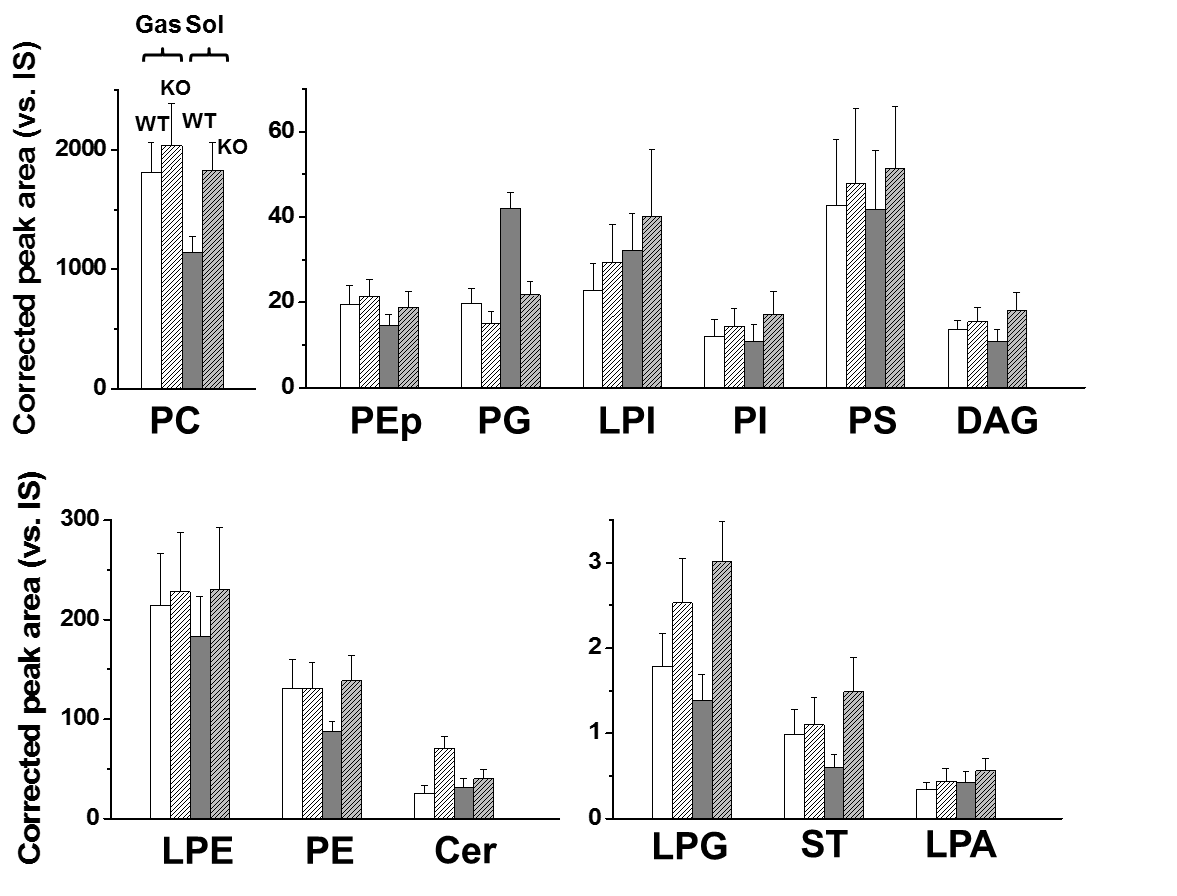


Figure S5. Total amounts (relative to IS of each lipid category) of 13 lipid categories (remaining part of Figure 3) in Gas and Sol tissues of WT and p53-KO mice, respectively.

Table S1. Identified/quantified numbers of lipids in each category from Gas and Sol tissues from p53 KO mice analyzed by nLC-ESI-MS/MS and types of precursor ion and SRM quantifier ion for each lipid class.

|  | Identified/quantified | Precursor  quantifier ion |
| --- | --- | --- |
| LPC | 12 | [M+H]^+^  [PCho+H]^+^ |
| PC | 46/26 | [M+H]^+^  [PCho+H]^+^ |
| LPE | 9 | [M+H]^+^  [M+H-141]^+^ |
| PE | 27/20 | [M+H]^+^  [M+H-141]^+^ |
| PEp | 24 | [M+H]^+^  [M+H-RCOOCH_2_CHCH_2_OH]^+^ ^a^ |
| DAG | 24 | [M+H]^+^  [M+ NH_4_]^+^ [M-RCOO]^+^ |
| TAG | 121/65 | [M+H]^+^  [M+ NH_4_]^+^ [M-RCOO]^+^ |
| SM | 10 | [M+H]^+^  [PCho+H]^+^ |
| Cer | 9 | [M+H]^+^  [d18:1]^+^ |
| MHC | 7 | [M+H]^+^  [d18:1]^+^ |
| ST | 8 | [M+H]^+^  [d18:1]^+^ |
| LPG | 5 | [M-H]^-^  [RCOO]^-^ |
| PG | 20 | [M-H]^-^  [RCOO]^-^ |
| LPI | 4 | [M-H]^-^  [RCOO]^-^ |
| PI | 30 | [M-H]^-^  [RCOO]^-^ |
| LPS | 5 | [M-H]^-^  [RCOO]^-^ |
| PS | 30 | [M-H]^-^  [RCOO]^-^ |
| LPA | 5 | [M-H]^-^  [RCOO]^-^ |
| PA | 16 | [M-H]^-^  [RCOO]^-^ |
| total | 412/329 |  |

^a^ Ref. for the SRM quantitation of PEp: Lee, S. T. et al. *Sci. Rep*. **6**, 36510-36520 (2016).

Table S2. Peak area (relative to I.S.) values of each lipid species in Gas and Sol tissue samples from WT and p53 KO mice and the ratio (KO/WT) along with the relative abundance (%) in each category based on WT mice. Bold species represents a significant change (*p* < 0.05) in p53 KO mice. Underlined species represents that existing in relatively high abundant in WT mice. Numbers followed by each lipid class are the quantified number of lipids in each category and those with asterisk (*) represent the numbers of total identified lipids with their molecular structures. Under the assumption that MS intensity of lipids is not significantly affected by the length and degree of unsaturation of acyl chains, peak area ratios can be considered as the relative values corresponding to 1 pmol of IS specific to each lipid class.

| Class | Molecular species | m/z | No | gastrocnemius | | | | soleus | | | |
| --- | --- | --- | --- | --- | --- | --- | --- | --- | --- | --- | --- |
|  |  |  |  | WT | KO | KO/WT | abun(%) | WT | KO | KO/WT | abun(%) |
| LPC | 14:0 | 468.3 |  | 0.09 ± 0.03 | 0.08 ± 0.02 | 0.89 ± 0.40 | 0.42 | 0.07 ± 0.02 | 0.14 ± 0.06 | 1.91 ± 0.93 | 0.36 |
| (12) | 16:0 | 496.3 | 1 | 4.75 ± 0.52 | 4.84 ± 0.65 | 1.02 ± 0.18 | 22.99 | 3.24 ± 0.31 | 7.31 ± 0.47 | 2.26 ± 0.26 | 16.14 |
|  | 16:1 | 494.4 |  | 0.23 ± 0.07 | 0.19 ± 0.07 | 0.82 ± 0.38 | 1.14 | 0.17 ± 0.05 | 0.35 ± 0.12 | 2.02 ± 0.94 | 0.86 |
|  | 18:0 | 524.4 | 2 | 2.62 ± 0.42 | 2.26 ± 0.23 | 0.86 ± 0.16 | 12.69 | 2.00 ± 0.51 | 4.12 ± 0.37 | 2.05 ± 0.55 | 10.00 |
|  | 18:1 | 522.3 |  | 1.18 ± 0.34 | 0.92 ± 0.17 | 0.78 ± 0.27 | 5.73 | 1.19 ± 0.37 | 2.70 ± 0.50 | 2.28 ± 0.83 | 5.91 |
|  | 18:2 | 520.3 | 3 | 1.82 ± 0.63 | 1.33 ± 0.24 | 0.73 ± 0.29 | 8.81 | 2.09 ± 0.82 | 6.68 ± 1.84 | 3.20 ± 1.54 | 10.42 |
|  | 20:3 | 546.4 |  | 0.16 ± 0.07 | 0.14 ± 0.03 | 0.84 ± 0.40 | 0.80 | 0.16 ± 0.04 | 0.46 ± 0.19 | 2.82 ± 1.29 | 0.82 |
|  | 20:4 | 544.3 | 4 | 0.68 ± 0.22 | 1.41 ± 0.48 | 2.09 ± 0.99 | 3.27 | 1.76 ± 0.66 | 0.92 ± 0.24 | 0.52 ± 0.24 | 8.76 |
|  | 22:0 | 580.4 |  | 0.95 ± 0.37 | 1.37 ± 0.75 | 1.45 ± 0.97 | 4.58 | 1.09 ± 0.24 | 1.87 ± 0.10 | 1.73 ± 0.39 | 5.42 |
|  | 22:4 | 572.4 |  | 0.10 ± 0.02 | 0.07 ± 0.01 | 0.67 ± 0.20 | 0.49 | 0.09 ± 0.03 | 0.23 ± 0.05 | 2.48 ± 0.96 | 0.45 |
|  | 22:5 | 570.4 |  | 0.80 ± 0.31 | 0.48 ± 0.08 | 0.60 ± 0.25 | 3.85 | 0.84 ± 0.19 | 3.28 ± 1.14 | 3.90 ± 1.61 | 4.20 |
|  | 22:6 | 568.3 | 5 | 7.28 ± 2.06 | 3.54 ± 0.85 | 0.49 ± 0.18 | 35.23 | 7.35 ± 3.50 | 18.26 ± 5.40 | 2.49 ± 1.39 | 36.66 |
| PC | **30:0** | 706.5 |  | 14.61 ± 2.99 | 18.21 ± 3.39 | 1.25 ± 0.35 | 0.81 | 11.31 ± 1.88 | 15.35 ± 3.77 | **1.36 ± 0.40** | 0.99 |
| (26  /46*) | **30:1** | 704.5 |  | 11.19 ± 1.40 | 11.84 ± 2.13 | 1.06 ± 0.23 | 0.62 | 6.79 ± 2.76 | 10.12 ± 1.10 | **1.49 ± 0.63** | 0.59 |
|  | 32:0 | 734.6 | 1 | 71.50 ± 7.91 | 79.43 ± 12.74 | 1.11 ± 0.22 | 3.96 | 53.06 ± 6.90 | 69.49 ± 9.40 | 1.31 ± 0.25 | 4.64 |
|  | 32:1 | 732.7 |  | 60.06 ± 9.36 | 75.50 ± 12.01 | 1.26 ± 0.28 | 3.32 | 32.57 ± 10.75 | 46.17 ± 7.31 | 1.42 ± 0.52 | 2.85 |
|  | 32:2 | 730.6 |  | 16.07 ± 2.16 | 17.10 ± 2.11 | 1.06 ± 0.19 | 0.89 | 8.70 ± 2.31 | 10.09 ± 0.93 | 1.16 ± 0.33 | 0.76 |
|  | **34:0** | 762.6 |  | 28.17 ± 2.95 | 33.49 ± 5.50 | 1.19 ± 0.23 | 1.56 | 21.64 ± 4.96 | 32.62 ± 3.95 | **1.51 ± 0.39** | 1.89 |
|  | **34:1** | 760.4 | 2 | 134.40 ± 18.06 | 154.73 ± 24.77 | 1.15 ± 0.24 | 7.44 | 86.45 ± 5.95 | 123.34 ± 18.71 | **1.43 ± 0.24** | 7.55 |
|  | **34:2** | 758.6 | 3 | 114.71 ± 15.86 | 130.07 ± 22.46 | 1.13 ± 0.25 | 6.35 | 65.65 ± 5.02 | 104.84 ± 11.64 | **1.60 ± 0.22** | 5.74 |
|  | 34:3 | 756.5 |  | 33.81 ± 6.27 | 42.85 ± 5.60 | 1.27 ± 0.29 | 1.87 | 19.33 ± 1.77 | 29.49 ± 4.01 | 1.53 ± 0.25 | 1.69 |
|  | 34:4 | 754.5 |  | 10.10 ± 1.86 | 10.61 ± 1.59 | 1.05 ± 0.25 | 0.56 | 4.51 ± 1.56 | 5.11 ± 0.54 | 1.13 ± 0.41 | 0.39 |
|  | **36:1** | 788.5 |  | 44.19 ± 4.55 | 53.65 ± 5.51 | 1.21 ± 0.18 | 2.45 | 41.17 ± 2.52 | 58.05 ± 5.49 | **1.41 ± 0.16** | 3.60 |
|  | 36:2 | 786.6 | 4 | 74.17 ± 8.92 | 80.22 ± 13.34 | 1.08 ± 0.22 | 4.11 | 62.31 ± 5.29 | 95.53 ± 11.03 | 1.53 ± 0.22 | 5.44 |
|  | 36:3 | 784.6 | 5 | 76.11 ± 11.45 | 105.59 ± 16.35 | 1.39 ± 0.30 | 4.21 | 42.79 ± 3.50 | 79.04 ± 12.00 | 1.85 ± 0.32 | 3.74 |
|  | **36:4** | 782.5 | 6 | 139.55 ± 21.03 | 163.67 ± 29.59 | 1.17 ± 0.28 | 7.72 | 74.86 ± 5.53 | 116.79 ± 11.72 | **1.56 ± 0.19** | 6.54 |
|  | 36:5 | 780.5 |  | 40.49 ± 5.09 | 47.22 ± 8.32 | 1.17 ± 0.25 | 2.24 | 21.06 ± 6.47 | 25.65 ± 3.10 | 1.22 ± 0.40 | 1.84 |
|  | 36:6 | 778.6 |  | 29.61 ± 6.01 | 26.63 ± 6.10 | 0.90 ± 0.27 | 1.64 | 13.24 ± 4.60 | 20.79 ± 3.51 | 1.57 ± 0.61 | 1.16 |
|  | 38:3 | 812.6 |  | 14.90 ± 2.23 | 17.02 ± 3.74 | 1.14 ± 0.30 | 0.82 | 13.88 ± 6.46 | 19.76 ± 4.34 | 1.42 ± 0.73 | 1.21 |
|  | **38:4** | 810.5 | 7 | 63.66 ± 7.71 | 76.04 ± 13.65 | 1.19 ± 0.26 | 3.52 | 51.09 ± 5.33 | 75.33 ± 8.49 | **1.47 ± 0.23** | 4.46 |
|  | 38:5 | 808.5 | 8 | 153.54 ± 21.23 | 172.54 ± 29.00 | 1.12 ± 0.24 | 8.50 | 92.71 ± 6.65 | 134.60 ± 17.64 | 1.45 ± 0.22 | 8.10 |
|  | **38:6** | 806.5 | 9 | 269.01 ± 37.12 | 290.42 ± 53.44 | 1.08 ± 0.25 | 14.89 | 155.64 ± 13.01 | 248.24 ± 28.02 | **1.59 ± 0.22** | 13.60 |
|  | **38:7** | 804.5 | 10 | 74.63 ± 10.88 | 83.43 ± 14.89 | 1.12 ± 0.26 | 4.13 | 40.58 ± 4.09 | 64.89 ± 7.23 | **1.60 ± 0.24** | 3.55 |
|  | **40:5** | 836.5 |  | 51.92 ± 4.62 | 49.05 ± 7.42 | 0.94 ± 0.17 | 2.87 | 36.77 ± 4.89 | 78.37 ± 12.25 | **2.13 ± 0.44** | 3.21 |
|  | 40:6 | 834.5 | 11 | 131.27 ± 24.14 | 137.45 ± 28.21 | 1.05 ± 0.29 | 7.27 | 97.54 ± 6.52 | 196.13 ± 27.28 | 2.01 ± 0.31 | 8.52 |
|  | 40:7 | 832.6 |  | 66.22 ± 10.16 | 81.72 ± 13.19 | 1.23 ± 0.27 | 3.67 | 40.02 ± 3.78 | 83.92 ± 9.92 | 2.10 ± 0.32 | 3.50 |
|  | 40:8 | 830.5 |  | 72.11 ± 10.24 | 66.72 ± 13.02 | 0.93 ± 0.22 | 3.99 | 45.95 ± 4.03 | 72.23 ± 7.87 | 1.57 ± 0.22 | 4.01 |
|  | **42:10** | 854.5 |  | 10.76 ± 2.08 | 11.12 ± 2.35 | 1.03 ± 0.30 | 0.60 | 4.93 ± 0.81 | 11.22 ± 2.02 | **2.28 ± 0.56** | 0.43 |
| LPE | 16:0 | 454.3 |  | 14.74 ± 2.54 | 12.44 ± 2.27 | 0.84 ± 0.21 | 6.88 | 10.04 ± 2.16 | 14.80 ± 3.36 | 1.47 ± 0.46 | 5.49 |
| (9) | 18:0 | 482.3 | 1 | 40.43 ± 6.82 | 35.61 ± 7.40 | 0.88 ± 0.24 | 18.86 | 35.52 ± 8.91 | 41.14 ± 8.74 | 1.16 ± 0.38 | 19.43 |
|  | 18:1 | 480.3 | 2 | 43.21 ± 12.41 | 33.69 ± 7.71 | 0.78 ± 0.29 | 20.16 | 26.80 ± 5.38 | 49.44 ± 13.93 | 1.84 ± 0.64 | 14.66 |
|  | 18:2 | 478.3 |  | 4.43 ± 1.09 | 4.97 ± 1.07 | 1.12 ± 0.37 | 2.07 | 4.34 ± 0.78 | 5.05 ± 0.97 | 1.16 ± 0.31 | 2.37 |
|  | 20:4 | 502.3 | 3 | 18.71 ± 2.53 | 11.01 ± 2.92 | 0.59 ± 0.18 | 8.73 | 22.41 ± 3.64 | 25.56 ± 4.17 | 1.14 ± 0.26 | 12.26 |
|  | 22:5 | 528.3 |  | 21.32 ± 5.43 | 21.56 ± 4.08 | 1.01 ± 0.32 | 9.95 | 16.14 ± 3.65 | 23.90 ± 5.06 | 1.48 ± 0.46 | 8.83 |
|  | 22:6 | 526.2 | 4 | 62.62 ± 19.33 | 100.30 ± 31.86 | 1.60 ± 0.71 | 29.21 | 63.26 ± 14.94 | 62.84 ± 23.92 | 0.99 ± 0.44 | 34.61 |
|  | 24:5 | 556.3 |  | 0.94 ± 0.34 | 1.23 ± 0.45 | 1.32 ± 0.67 | 0.44 | 0.56 ± 0.26 | 1.18 ± 0.42 | 2.12 ± 1.22 | 0.31 |
|  | **24:6** | 554.3 |  | 7.97 ± 1.83 | 7.08 ± 1.68 | 0.89 ± 0.29 | 3.72 | 3.73 ± 0.91 | 6.75 ± 1.22 | **1.81 ± 0.55** | 2.04 |
| PE | **34:0** | 720.6 |  | 1.54 ± 0.10 | 1.70 ± 0.09 | 1.10 ± 0.09 | 1.18 | 1.01 ± 0.03 | 1.82 ± 0.11 | **1.80 ± 0.12** | 1.16 |
| (20  /27*) | 34:1 | 718.5 |  | 1.33 ± 0.23 | 1.50 ± 0.36 | 1.13 ± 0.33 | 1.01 | 1.03 ± 0.32 | 1.37 ± 0.24 | 1.33 ± 0.47 | 1.18 |
|  | 36:1 | 746.6 |  | 1.49 ± 0.16 | 2.10 ± 0.16 | 1.40 ± 0.18 | 1.14 | 1.51 ± 0.08 | 1.94 ± 0.12 | 1.28 ± 0.11 | 1.73 |
|  | 36:2 | 744.5 |  | 3.79 ± 0.79 | 4.15 ± 0.44 | 1.10 ± 0.26 | 2.89 | 3.21 ± 0.33 | 4.93 ± 0.91 | 1.54 ± 0.32 | 3.68 |
|  | 36:3 | 742.6 |  | 1.06 ± 0.16 | 0.98 ± 0.17 | 0.92 ± 0.22 | 0.81 | 0.73 ± 0.20 | 1.10 ± 0.19 | 1.50 ± 0.48 | 0.84 |
|  | 36:4 | 740.6 |  | 1.59 ± 0.48 | 1.24 ± 0.28 | 0.78 ± 0.29 | 1.21 | 0.89 ± 0.17 | 1.16 ± 0.16 | 1.30 ± 0.30 | 1.02 |
|  | **36:6** | 736.6 |  | 0.46 ± 0.09 | 0.39 ± 0.13 | 0.86 ± 0.34 | 0.35 | 0.14 ± 0.04 | 0.23 ± 0.07 | **1.71 ± 0.74** | 0.16 |
|  | **36:7** | 734.6 |  | 0.08 ± 0.01 | 0.08 ± 0.01 | 1.03 ± 0.17 | 0.06 | 0.05 ± 0.01 | 0.09 ± 0.00 | **1.90 ± 0.36** | 0.05 |
|  | 38:4 | 768.5 | 1 | 8.50 ± 2.48 | 9.23 ± 1.83 | 1.09 ± 0.38 | 6.48 | 4.99 ± 0.8 | 11.73 ± 3.72 | 2.35 ± 0.84 | 5.72 |
|  | **38:5** | 766.6 |  | 5.08 ± 0.93 | 5.19 ± 0.73 | 1.02 ± 0.24 | 3.87 | 2.30 ± 0.26 | 4.78 ± 0.44 | **2.08 ± 0.30** | 2.64 |
|  | 38:6 | 764.5 | 2 | 21.39 ± 5.67 | 17.82 ± 3.61 | 0.83 ± 0.28 | 16.31 | 13.34 ± 2.36 | 17.68 ± 2.54 | 1.33 ± 0.30 | 15.28 |
|  | 38:7 | 762.4 |  | 2.72 ± 0.71 | 2.27 ± 0.25 | 0.84 ± 0.24 | 2.07 | 1.22 ± 0.37 | 1.75 ± 0.24 | 1.44 ± 0.48 | 1.40 |
|  | 40:5 | 794.5 | 3 | 12.37 ± 2.64 | 13.84 ± 3.64 | 1.12 ± 0.38 | 9.43 | 8.07 ± 0.92 | 14.73 ± 4.54 | 1.82 ± 0.60 | 9.25 |
|  | 40:6 | 792.6 | 4 | 46.74 ± 8.48 | 50.36 ± 10.79 | 1.08 ± 0.30 | 35.64 | 34.50 ± 2.70 | 54.49 ± 10.55 | 1.58 ± 0.33 | 39.53 |
|  | 40:7 | 790.5 | 5 | 11.60 ± 2.71 | 10.09 ± 1.38 | 0.87 ± 0.24 | 8.85 | 8.08 ± 1.19 | 12.07 ± 0.67 | 1.49 ± 0.24 | 9.26 |
|  | 40:8 | 788.5 | 6 | 8.19 ± 2.27 | 6.64 ± 0.99 | 0.81 ± 0.26 | 6.24 | 4.26 ± 0.74 | 6.45 ± 0.67 | 1.51 ± 0.31 | 4.88 |
|  | 40:12 | 780.4 |  | 0.61 ± 0.08 | 0.63 ± 0.11 | 1.04 ± 0.23 | 0.46 | 0.51 ± 0.07 | 0.75 ± 0.11 | 1.47 ± 0.30 | 0.59 |
|  | 42:12 | 808.5 |  | 0.24 ± 0.03 | 0.28 ± 0.04 | 1.19 ± 0.23 | 0.18 | 0.30 ± 0.05 | 0.33 ± 0.05 | 1.11 ± 0.26 | 0.34 |
|  | 44:10 | 840.6 |  | 1.38 ± 0.22 | 1.58 ± 0.33 | 1.15 ± 0.30 | 1.05 | 0.63 ± 0.15 | 0.91 ± 0.23 | 1.43 ± 0.50 | 0.73 |
|  | 44:11 | 838.5 |  | 1.01 ± 0.22 | 1.17 ± 0.21 | 1.16 ± 0.33 | 0.77 | 0.50 ± 0.05 | 0.44 ± 0.10 | 0.88 ± 0.23 | 0.57 |
| PEp | 16:0p/18:1 | 702.5 | 1 | 0.86 ± 0.13 | 0.97 ± 0.13 | 1.13 ± 0.23 | 4.39 | 0.96 ± 0.33 | 1.01 ± 0.16 | 1.04 ± 0.40 | 6.61 |
| (24) | 16:0p/20:1 | 730.5 |  | 0.03 ± 0.00 | 0.04 ± 0.00 | 1.28 ± 0.22 | 0.14 | 0.03 ± 0.00 | 0.05 ± 0.01 | 1.72 ± 0.36 | 0.18 |
|  | 16:0p/20:4 | 724.5 |  | 0.61 ± 0.17 | 0.65 ± 0.21 | 1.06 ± 0.45 | 3.13 | 0.35 ± 0.06 | 0.53 ± 0.11 | 1.52 ± 0.41 | 2.38 |
|  | 16:0p/22:4 | 750.6 | 2 | 1.00 ± 0.23 | 1.06 ± 0.21 | 1.06 ± 0.33 | 5.10 | 0.56 ± 0.09 | 0.73 ± 0.11 | 1.30 ± 0.28 | 3.86 |
|  | 16:0p/22:6 | 748.5 | 3 | 5.76 ± 1.50 | 5.53 ± 1.26 | 0.96 ± 0.33 | 29.40 | 3.14 ± 0.91 | 4.63 ± 1.23 | 1.47 ± 0.58 | 21.53 |
|  | 16:0p/24:5 | 778.5 |  | 0.01 ± 0.00 | 0.01 ± 0.00 | 1.05 ± 0.26 | 0.04 | 0.01 ± 0.00 | 0.01 ± 0.00 | 1.55 ± 0.38 | 0.05 |
|  | 18:0p/18:0 | 732.5 | 4 | 0.10 ± 0.02 | 0.15 ± 0.02 | 1.42 ± 0.33 | 0.53 | 0.12 ± 0.02 | 0.13 ± 0.03 | 1.12 ± 0.27 | 0.82 |
|  | 18:0p/18:1 | 730.5 |  | 2.77 ± 0.46 | 3.88 ± 0.53 | 1.40 ± 0.30 | 14.12 | 2.98 ± 0.27 | 3.18 ± 0.49 | 1.07 ± 0.19 | 20.40 |
|  | **18:0p/18:2** | 728.5 |  | 0.22 ± 0.06 | 0.23 ± 0.02 | 1.04 ± 0.30 | 1.12 | 0.13 ± 0.01 | 0.30 ± 0.04 | **2.27 ± 0.34** | 0.91 |
|  | **18:0p/20:1** | 758.5 |  | 0.02 ± 0.00 | 0.03 ± 0.00 | 1.35 ± 0.22 | 0.12 | 0.02 ± 0.00 | 0.03 ± 0.00 | **2.13 ± 0.65** | 0.11 |
|  | **18:0p/20:4** | 752.6 |  | 0.59 ± 0.13 | 0.66 ± 0.12 | 1.12 ± 0.33 | 3.01 | 0.32 ± 0.04 | 0.66 ± 0.06 | **2.05 ± 0.32** | 2.20 |
|  | **18:0p/22:5** | 778.5 |  | 0.77 ± 0.14 | 0.89 ± 0.11 | 1.16 ± 0.26 | 3.92 | 0.38 ± 0.05 | 0.71 ± 0.09 | **1.84 ± 0.33** | 2.63 |
|  | 18:0p/24:5 | 806.5 |  | 0.01 ± 0.00 | 0.01 ± 0.00 | 1.61 ± 0.48 | 0.04 | 0.01 ± 0.00 | 0.01 ± 0.00 | 1.60 ± 0.40 | 0.05 |
|  | 18:1p/16:0 | 702.5 |  | 0.51 ± 0.08 | 0.57 ± 0.07 | 1.12 ± 0.24 | 2.58 | 0.37 ± 0.04 | 0.56 ± 0.13 | 1.49 ± 0.39 | 2.56 |
|  | 18:1p/18:0 | 730.5 |  | 0.11 ± 0.02 | 0.15 ± 0.02 | 1.33 ± 0.27 | 0.58 | 0.13 ± 0.01 | 0.14 ± 0.02 | 1.01 ± 0.19 | 0.92 |
|  | 18:1p/18:1 | 728.5 | 5 | 3.70 ± 0.86 | 4.03 ± 0.50 | 1.09 ± 0.29 | 18.88 | 3.54 ± 0.39 | 3.67 ± 0.78 | 1.04 ± 0.25 | 24.24 |
|  | 18:1p/20:4 | 750.6 |  | 0.28 ± 0.06 | 0.35 ± 0.09 | 1.23 ± 0.42 | 1.44 | 0.20 ± 0.04 | 0.25 ± 0.06 | 1.21 ± 0.35 | 1.39 |
|  | **18:1p/22:4** | 778.5 |  | 0.13 ± 0.03 | 0.17 ± 0.02 | 1.31 ± 0.39 | 0.65 | 0.07 ± 0.01 | 0.14 ± 0.02 | **1.91 ± 0.41** | 0.49 |
|  | 18:1p/22:6 | 774.6 | 6 | 2.03 ± 0.46 | 1.98 ± 0.49 | 0.98 ± 0.33 | 10.37 | 1.19 ± 0.27 | 2.00 ± 0.42 | 1.68 ± 0.53 | 8.14 |
|  | 18:1p/24:4 | 806.5 |  | 0.01 ± 0.00 | 0.02 ± 0.00 | 1.45 ± 0.39 | 0.07 | 0.02 ± 0.00 | 0.01 ± 0.01 | 0.91 ± 0.41 | 0.10 |
|  | 20:0p/18:1 | 758.5 |  | 0.04 ± 0.00 | 0.05 ± 0.00 | 1.40 ± 0.18 | 0.18 | 0.03 ± 0.01 | 0.05 ± 0.00 | 1.85 ± 0.62 | 0.18 |
|  | 20:0p/22:5 | 806.5 |  | 0.02 ± 0.00 | 0.02 ± 0.00 | 1.23 ± 0.22 | 0.10 | 0.02 ± 0.00 | 0.02 ± 0.01 | 1.11 ± 0.28 | 0.14 |
|  | 20:1p/20:4 | 778.6 |  | 0.01 ± 0.00 | 0.02 ± 0.00 | 1.16 ± 0.30 | 0.07 | 0.01 ± 0.00 | 0.02 ± 0.00 | 1.33 ± 0.28 | 0.08 |
|  | 20:1p/22:4 | 806.6 |  | 0.01 ± 0.00 | 0.01 ± 0.00 | 1.21 ± 0.39 | 0.03 | 0.00 ± 0.00 | 0.01 ± 0.00 | 1.89 ± 0.55 | 0.03 |
| LPG | 14:0 | 455.4 |  | 0.01 ± 0.00 | 0.02 ± 0.01 | 1.26 ± 0.45 | 0.82 | 0.01 ± 0.00 | 0.03 ± 0.01 | 1.81 ± 0.75 | 1.00 |
| (5) | 16:0 | 483.3 |  | 0.07 ± 0.01 | 0.09 ± 0.02 | 1.36 ± 0.37 | 3.77 | 0.14 ± 0.02 | 0.20 ± 0.02 | 1.41 ± 0.28 | 10.10 |
|  | 18:0 | 511.4 |  | 0.25 ± 0.03 | 0.31 ± 0.04 | 1.23 ± 0.21 | 13.92 | 0.19 ± 0.03 | 0.35 ± 0.04 | 1.90 ± 0.35 | 13.43 |
|  | 18:1 | 509.3 | 1 | 1.38 ± 0.32 | 1.92 ± 0.41 | 1.40 ± 0.44 | 76.84 | 0.86 ± 0.20 | 2.11 ± 0.36 | 2.46 ± 0.70 | 61.89 |
|  | 18:2 | 507.2 |  | 0.08 ± 0.01 | 0.19 ± 0.05 | 2.30 ± 0.68 | 4.64 | 0.19 ± 0.05 | 0.32 ± 0.05 | 1.67 ± 0.47 | 13.59 |
| PG | 16:0/14:0 | 693.5 |  | 0.18 ± 0.02 | 0.18 ± 0.01 | 0.98 ± 0.12 | 0.90 | 0.16 ± 0.02 | 0.16 ± 0.03 | 1.03 ± 0.23 | 0.38 |
| (20) | 16:0/16:0 | 721.5 | 1 | 0.87 ± 0.11 | 0.50 ± 0.08 | 0.57 ± 0.12 | 4.38 | 0.78 ± 0.05 | 0.61 ± 0.12 | 0.79 ± 0.16 | 1.84 |
|  | 16:0/16:1 | 719.5 |  | 0.17 ± 0.09 | 0.24 ± 0.05 | 1.42 ± 0.78 | 0.86 | 0.30 ± 0.04 | 0.28 ± 0.01 | 0.94 ± 0.14 | 0.71 |
|  | 18:0/16:0 | 749.5 |  | 0.41 ± 0.06 | 0.25 ± 0.06 | 0.59 ± 0.16 | 2.09 | 0.88 ± 0.09 | 0.37 ± 0.06 | 0.42 ± 0.08 | 2.08 |
|  | 18:0/22:6 | 821.5 |  | 0.24 ± 0.07 | 0.15 ± 0.04 | 0.60 ± 0.26 | 1.22 | 1.21 ± 0.14 | 0.31 ± 0.05 | 0.26 ± 0.05 | 2.87 |
|  | 18:1/14:0 | 719.5 |  | 0.10 ± 0.03 | 0.09 ± 0.01 | 0.89 ± 0.28 | 0.51 | 0.09 ± 0.02 | 0.06 ± 0.01 | 0.61 ± 0.15 | 0.22 |
|  | 18:1/16:0 | 747.6 | 2 | 9.27 ± 1.52 | 6.24 ± 1.27 | 0.67 ± 0.18 | 46.71 | 19.67 ± 1.46 | 8.25 ± 1.17 | 0.42 ± 0.07 | 46.66 |
|  | 18:1/16:1 | 745.6 | 3 | 2.21 ± 0.37 | 1.50 ± 0.14 | 0.68 ± 0.13 | 11.16 | 1.28 ± 0.14 | 2.35 ± 0.28 | 1.83 ± 0.30 | 3.05 |
|  | 18:1/18:0 | 775.5 | 4 | 0.55 ± 0.11 | 0.46 ± 0.07 | 0.82 ± 0.21 | 2.78 | 3.23 ± 0.38 | 0.90 ± 0.12 | 0.28 ± 0.05 | 7.67 |
|  | 18:1/18:1 | 773.6 |  | 0.57 ± 0.17 | 0.60 ± 0.14 | 1.06 ± 0.40 | 2.85 | 1.94 ± 0.14 | 0.77 ± 0.14 | 0.40 ± 0.08 | 4.60 |
|  | 18:1/18:2 | 771.7 | 5 | 1.47 ± 0.19 | 1.70 ± 0.18 | 1.16 ± 0.19 | 7.39 | 4.90 ± 0.37 | 2.98 ± 0.37 | 0.61 ± 0.09 | 11.62 |
|  | 18:2/16:0 | 745.6 | 6 | 1.39 ± 0.28 | 1.21 ± 0.22 | 0.87 ± 0.23 | 7.02 | 1.56 ± 0.23 | 1.23 ± 0.26 | 0.79 ± 0.20 | 3.70 |
|  | 18:2/22:6 | 817.5 |  | 0.17 ± 0.01 | 0.13 ± 0.03 | 0.76 ± 0.21 | 0.86 | 0.18 ± 0.03 | 0.33 ± 0.03 | 1.8 ± 0.29 | 0.43 |
|  | 20:1/16:0 | 775.5 |  | 0.17 ± 0.02 | 0.13 ± 0.04 | 0.81 ± 0.27 | 0.84 | 0.98 ± 0.09 | 0.46 ± 0.06 | 0.48 ± 0.08 | 2.32 |
|  | 20:2/16:0 | 773.5 | 7 | 0.67 ± 0.16 | 0.49 ± 0.09 | 0.73 ± 0.23 | 3.36 | 3.10 ± 0.12 | 0.90 ± 0.22 | 0.29 ± 0.07 | 7.36 |
|  | 20:3/16:0 | 771.7 |  | 0.07 ± 0.01 | 0.04 ± 0.00 | 0.60 ± 0.13 | 0.34 | 0.12 ± 0.03 | 0.10 ± 0.01 | 0.89 ± 0.23 | 0.28 |
|  | 22:5/16:0 | 795.5 |  | 0.22 ± 0.03 | 0.18 ± 0.04 | 0.84 ± 0.21 | 1.10 | 0.42 ± 0.08 | 0.34 ± 0.05 | 0.81 ± 0.19 | 0.99 |
|  | 22:6/16:0 | 793.5 |  | 0.55 ± 0.09 | 0.47 ± 0.09 | 0.86 ± 0.22 | 2.76 | 0.39 ± 0.06 | 0.68 ± 0.04 | 1.73 ± 0.29 | 0.93 |
|  | 22:6/18:1 | 819.5 |  | 0.42 ± 0.09 | 0.44 ± 0.06 | 1.06 ± 0.26 | 2.10 | 0.59 ± 0.10 | 0.44 ± 0.06 | 0.75 ± 0.16 | 1.40 |
|  | 22:6/22:6 | 865.5 |  | 0.15 ± 0.06 | 0.11 ± 0.05 | 0.77 ± 0.47 | 0.75 | 0.37 ± 0.09 | 0.23 ± 0.04 | 0.63 ± 0.19 | 0.88 |
| LPI | 16:0 | 571.3 |  | 4.89 ± 0.78 | 5.65 ± 1.11 | 1.16 ± 0.29 | 21.37 | 7.52 ± 1.94 | 8.31 ± 1.63 | 1.11 ± 0.36 | 23.42 |
| (4) | 18:0 | 599.4 | 1 | 12.71 ± 4.06 | 14.84 ± 5.60 | 1.17 ± 0.58 | 55.56 | 17.19 ± 5.23 | 23.71 ± 6.51 | 1.38 ± 0.57 | 53.54 |
|  | **20:4** | 619.4 |  | 4.45 ± 1.27 | 7.92 ± 1.82 | **1.78 ± 0.65** | 19.46 | 6.04 ± 1.34 | 6.24 ± 2.05 | 1.03 ± 0.41 | 18.81 |
|  | **22:6** | 643.3 |  | 0.83 ± 0.23 | 1.07 ± 0.16 | **1.30 ± 0.41** | 3.62 | 1.36 ± 0.23 | 1.94 ± 0.34 | 1.42 ± 0.35 | 4.23 |
| PI | 16:0/16:0 | 809.5 |  | 0.14 ± 0.04 | 0.13 ± 0.03 | 0.98 ± 0.37 | 1.27 | 0.15 ± 0.04 | 0.18 ± 0.05 | 1.22 ± 0.49 | 1.48 |
| (30) | 16:0/16:1 | 807.5 |  | 0.16 ± 0.02 | 0.22 ± 0.05 | 1.35 ± 0.34 | 1.50 | 0.18 ± 0.05 | 0.24 ± 0.08 | 1.29 ± 0.55 | 1.84 |
|  | 16:0/20:3 | 859.6 |  | 0.03 ± 0.01 | 0.03 ± 0.01 | 1.04 ± 0.43 | 0.27 | 0.04 ± 0.02 | 0.03 ± 0.01 | 0.60 ± 0.29 | 0.43 |
|  | 16:0/22:5 | 883.5 | 1 | 0.37 ± 0.15 | 0.41 ± 0.11 | 1.12 ± 0.54 | 3.39 | 0.22 ± 0.09 | 0.29 ± 0.07 | 1.31 ± 0.61 | 2.18 |
|  | 18:0/14:0 | 809.5 |  | 0.09 ± 0.03 | 0.06 ± 0.02 | 0.73 ± 0.32 | 0.81 | 0.10 ± 0.05 | 0.11 ± 0.03 | 1.04 ± 0.54 | 1.03 |
|  | **18:0/16:0** | 837.5 | 2 | 0.67 ± 0.12 | 0.57 ± 0.16 | 0.85 ± 0.29 | 6.21 | 0.61 ± 0.11 | 1.12 ± 0.38 | **1.82 ± 0.70** | 6.11 |
|  | 18:0/16:1 | 835.7 |  | 0.07 ± 0.03 | 0.05 ± 0.01 | 0.65 ± 0.28 | 0.68 | 0.04 ± 0.00 | 0.06 ± 0.01 | 1.52 ± 0.37 | 0.43 |
|  | 18:0/16:2 | 833.5 |  | 0.16 ± 0.05 | 0.11 ± 0.03 | 0.70 ± 0.29 | 1.48 | 0.19 ± 0.05 | 0.23 ± 0.08 | 1.21 ± 0.53 | 1.86 |
|  | **18:0/18:0** | 865.5 |  | 0.08 ± 0.03 | 0.06 ± 0.02 | 0.74 ± 0.34 | 0.71 | 0.04 ± 0.01 | 0.12 ± 0.05 | **2.94 ± 1.23** | 0.41 |
|  | 18:0/18:1 | 863.5 |  | 0.22 ± 0.06 | 0.24 ± 0.08 | 1.11 ± 0.49 | 2.01 | 0.21 ± 0.07 | 0.33 ± 0.10 | 1.54 ± 0.67 | 2.14 |
|  | 18:0/18:2 | 861.5 | 3 | 1.03 ± 0.41 | 0.75 ± 0.18 | 0.72 ± 0.34 | 9.59 | 1.09 ± 0.29 | 1.69 ± 0.56 | 1.55 ± 0.66 | 10.89 |
|  | **18:0/20:2** | 889.6 |  | 0.20 ± 0.07 | 0.11 ± 0.02 | 0.53 ± 0.21 | 1.94 | 0.09 ± 0.02 | 0.16 ± 0.05 | **1.72 ± 0.68** | 0.91 |
|  | 18:0/20:3 | 887.7 | 4 | 1.02 ± 0.45 | 0.58 ± 0.15 | 0.56 ± 0.29 | 9.48 | 0.52 ± 0.20 | 0.62 ± 0.15 | 1.18 ± 0.53 | 5.23 |
|  | **18:0/20:4** | 885.6 | 5 | 3.40 ± 0.90 | 6.64 ± 1.41 | **1.96 ± 0.66** | 31.52 | 4.08 ± 1.06 | 6.02 ± 1.28 | **1.47 ± 0.50** | 40.78 |
|  | **18:0/20:5** | 883.5 |  | 0.11 ± 0.03 | 0.05 ± 0.01 | **0.48 ± 0.16** | 1.02 | 0.07 ± 0.02 | 0.12 ± 0.04 | 1.62 ± 0.63 | 0.73 |
|  | 18:0/22:4 | 913.6 |  | 0.12 ± 0.04 | 0.12 ± 0.05 | 1.02 ± 0.52 | 1.07 | 0.07 ± 0.03 | 0.10 ± 0.03 | 1.46 ± 0.73 | 0.67 |
|  | 18:0/22:5 | 911.5 | 6 | 0.74 ± 0.29 | 0.53 ± 0.16 | 0.72 ± 0.36 | 6.88 | 0.34 ± 0.12 | 0.54 ± 0.18 | 1.57 ± 0.76 | 3.43 |
|  | 18:0/22:6 | 909.5 | 7 | 0.73 ± 0.27 | 0.67 ± 0.21 | 0.93 ± 0.45 | 6.76 | 0.66 ± 0.35 | 1.03 ± 0.37 | 1.54 ± 0.99 | 6.63 |
|  | 18:1/14:0 | 807.5 |  | 0.12 ± 0.02 | 0.19 ± 0.07 | 1.58 ± 0.67 | 1.12 | 0.12 ± 0.03 | 0.18 ± 0.05 | 1.53 ± 0.56 | 1.20 |
|  | 18:1/16:0 | 835.7 |  | 0.13 ± 0.04 | 0.13 ± 0.03 | 1.01 ± 0.38 | 1.21 | 0.14 ± 0.05 | 0.21 ± 0.08 | 1.43 ± 0.70 | 1.45 |
|  | 18:1/16:1 | 833.5 |  | 0.11 ± 0.02 | 0.10 ± 0.04 | 0.87 ± 0.40 | 1.05 | 0.11 ± 0.04 | 0.13 ± 0.02 | 1.14 ± 0.41 | 1.12 |
|  | 18:1/18:1 | 861.5 |  | 0.05 ± 0.01 | 0.08 ± 0.02 | 1.51 ± 0.47 | 0.49 | 0.06 ± 0.01 | 0.05 ± 0.02 | 0.96 ± 0.36 | 0.56 |
|  | 18:1/18:2 | 859.6 |  | 0.08 ± 0.02 | 0.10 ± 0.03 | 1.17 ± 0.39 | 0.76 | 0.12 ± 0.03 | 0.15 ± 0.03 | 1.30 ± 0.41 | 1.19 |
|  | 18:1/20:2 | 887.7 |  | 0.03 ± 0.01 | 0.04 ± 0.01 | 1.33 ± 0.63 | 0.25 | 0.02 ± 0.01 | 0.04 ± 0.01 | 1.64 ± 0.86 | 0.22 |
|  | 18:1/20:3 | 885.6 |  | 0.06 ± 0.01 | 0.08 ± 0.02 | 1.26 ± 0.41 | 0.55 | 0.03 ± 0.01 | 0.03 ± 0.01 | 0.97 ± 0.39 | 0.29 |
|  | **18:1/20:4** | 883.5 | 8 | 0.55 ± 0.19 | 1.14 ± 0.38 | **2.09 ± 1.01** | 5.06 | 0.45 ± 0.17 | 0.58 ± 0.22 | 1.29 ± 0.69 | 4.51 |
|  | 18:1/22:5 | 909.5 |  | 0.12 ± 0.05 | 0.11 ± 0.03 | 0.94 ± 0.51 | 1.07 | 0.05 ± 0.01 | 0.10 ± 0.03 | 1.80 ± 0.77 | 0.54 |
|  | **18:1/22:6** | 907.6 |  | 0.09 ± 0.02 | 0.15 ± 0.05 | **1.69 ± 0.71** | 0.81 | 0.12 ± 0.04 | 0.14 ± 0.04 | 1.09 ± 0.51 | 1.24 |
|  | **18:2/16:0** | 833.5 |  | 0.10 ± 0.03 | 0.07 ± 0.01 | **0.69 ± 0.23** | 0.91 | 0.05 ± 0.02 | 0.07 ± 0.02 | 1.44 ± 0.62 | 0.47 |
|  | **20:4/20:4** | 905.5 |  | 0.01 ± 0.00 | 0.02 ± 0.01 | 1.18 ± 0.65 | 0.12 | 0.01 ± 0.00 | 0.02 ± 0.01 | **1.50 ± 0.76** | 0.11 |
| LPS | 14:0 | 468.3 |  | 0.21 ± 0.04 | 0.31 ± 0.09 | 1.44 ± 0.51 | 0.333 | 0.21 ± 0.05 | 0.23 ± 0.07 | 1.12 ± 0.41 | 0.43 |
| (5) | **18:0** | 524.4 | 1 | 36.41 ± 5.01 | 42.37 ± 7.15 | 1.16 ± 0.25 | 56.53 | 23.84 ± 3.70 | 38.38 ± 4.12 | **1.61 ± 0.30** | 49.18 |
|  | **18:1** | 522.3 |  | 9.88 ± 1.09 | 13.42 ± 1.00 | **1.36 ± 0.18** | 15.33 | 7.97 ± 0.58 | 12.42 ± 1.56 | **1.56 ± 0.23** | 16.44 |
|  | 20:3 | 546.4 |  | 4.68 ± 0.73 | 4.94 ± 0.80 | 1.06 ± 0.24 | 7.26 | 4.25 ± 0.48 | 4.93 ± 0.67 | 1.16 ± 0.20 | 8.78 |
|  | 22:6 | 568.3 | 2 | 13.24 ± 1.73 | 17.10 ± 1.34 | 1.29 ± 0.20 | 20.55 | 12.21 ± 1.57 | 16.37 ± 1.46 | 1.34 ± 0.21 | 25.18 |
| PS | 16:0/18:1 | 760.7 |  | 0.09 ± 0.03 | 0.09 ± 0.03 | 1.01 ± 0.45 | 0.21 | 0.07 ± 0.01 | 0.06 ± 0.02 | 0.91 ± 0.30 | 0.15 |
| (30) | 16:0/22:4 | 810.6 |  | 0.23 ± 0.09 | 0.19 ± 0.05 | 0.81 ± 0.37 | 0.55 | 0.19 ± 0.05 | 0.21 ± 0.06 | 1.12 ± 0.44 | 0.50 |
|  | 16:0/22:5 | 808.6 |  | 0.16 ± 0.05 | 0.14 ± 0.04 | 0.86 ± 0.35 | 0.38 | 0.14 ± 0.04 | 0.18 ± 0.04 | 1.27 ± 0.47 | 0.44 |
|  | 16:0/22:6 | 806.6 |  | 1.14 ± 0.35 | 1.62 ± 0.61 | 1.43 ± 0.69 | 2.67 | 1.17 ± 0.42 | 1.10 ± 0.20 | 0.94 ± 0.37 | 2.64 |
|  | **16:0/24:4** | 838.6 |  | 0.12 ± 0.02 | 0.08 ± 0.02 | **0.67 ± 0.22** | 0.28 | 0.09 ± 0.01 | 0.13 ± 0.03 | 1.42 ± 0.39 | 0.32 |
|  | 16:0/24:5 | 836.6 |  | 0.17 ± 0.06 | 0.18 ± 0.05 | 1.04 ± 0.47 | 0.40 | 0.11 ± 0.04 | 0.17 ± 0.06 | 1.46 ± 0.70 | 0.40 |
|  | 18:0/16:0 | 762.6 | 1 | 2.79 ± 0.78 | 3.12 ± 0.64 | 1.12 ± 0.39 | 6.55 | 3.92 ± 1.38 | 3.15 ± 0.94 | 0.80 ± 0.37 | 7.55 |
|  | 18:0/16:1 | 760.7 |  | 0.08 ± 0.02 | 0.12 ± 0.04 | 1.46 ± 0.64 | 0.19 | 0.08 ± 0.05 | 0.09 ± 0.02 | 1.19 ± 0.78 | 0.23 |
|  | 18:0/18:0 | 790.6 | 2 | 13.31 ± 6.14 | 10.46 ± 7.74 | 0.79 ± 0.68 | 31.23 | 9.94 ± 3.73 | 17.96 ± 4.15 | 1.81 ± 0.80 | 43.04 |
|  | 18:0/18:1 | 788.7 | 3 | 2.04 ± 0.35 | 2.02 ± 0.51 | 0.99 ± 0.30 | 4.78 | 1.76 ± 0.52 | 1.98 ± 0.64 | 1.13 ± 0.49 | 4.74 |
|  | **18:0/18:2** | 786.7 |  | 0.78 ± 0.24 | 1.14 ± 0.30 | **1.46 ± 0.60** | 1.82 | 0.74 ± 0.31 | 0.44 ± 0.07 | 0.59 ± 0.26 | 1.05 |
|  | **18:0/20:0** | 818.7 | 4 | 1.14 ± 0.34 | 1.54 ± 0.42 | **1.35 ± 0.55** | 2.68 | 1.69 ± 0.51 | 1.72 ± 0.60 | 1.02 ± 0.47 | 4.13 |
|  | 18:0/20:4 | 810.6 |  | 0.32 ± 0.10 | 0.30 ± 0.08 | 0.95 ± 0.38 | 0.75 | 0.21 ± 0.06 | 0.29 ± 0.08 | 1.34 ± 0.50 | 0.69 |
|  | 18:0/20:5 | 808.6 |  | 0.21 ± 0.06 | 0.18 ± 0.07 | 0.87 ± 0.41 | 0.49 | 0.15 ± 0.05 | 0.19 ± 0.06 | 1.29 ± 0.59 | 0.45 |
|  | **18:0/22:4** | 838.6 |  | 0.09 ± 0.03 | 0.19 ± 0.08 | **2.05 ± 1.18** | 0.21 | 0.13 ± 0.05 | 0.06 ± 0.02 | **0.47 ± 0.23** | 0.15 |
|  | 18:0/22:6 | 834.6 | 5 | 5.67 ± 2.55 | 9.65 ± 1.90 | 1.70 ± 0.84 | 13.31 | 4.05 ± 1.42 | 5.19 ± 1.68 | 1.28 ± 0.61 | 12.45 |
|  | 18:0/24:4 | 866.6 |  | 0.25 ± 0.09 | 0.31 ± 0.16 | 1.22 ± 0.76 | 0.59 | 0.20 ± 0.07 | 0.21 ± 0.06 | 1.06 ± 0.49 | 0.50 |
|  | 18:0/24:6 | 862.5 |  | 0.42 ± 0.17 | 0.36 ± 0.12 | 0.86 ± 0.45 | 0.99 | 0.43 ± 0.14 | 0.58 ± 0.16 | 1.34 ± 0.59 | 1.39 |
|  | 18:1/22:4 | 836.6 |  | 0.15 ± 0.05 | 0.18 ± 0.08 | 1.16 ± 0.69 | 0.36 | 0.08 ± 0.02 | 0.12 ± 0.03 | 1.43 ± 0.50 | 0.39 |
|  | 18:1/22:6 | 832.6 | 6 | 3.35 ± 1.03 | 3.76 ± 1.06 | 1.12 ± 0.47 | 7.85 | 3.94 ± 1.25 | 4.75 ± 1.50 | 1.21 ± 0.54 | 11.38 |
|  | 18:2/22:6 | 830.4 | 7 | 3.63 ± 1.09 | 3.13 ± 0.71 | 0.86 ± 0.33 | 8.52 | 4.17 ± 1.18 | 4.46 ± 1.47 | 1.07 ± 0.46 | 10.70 |
|  | 20:0/22:6 | 862.5 |  | 0.06 ± 0.02 | 0.05 ± 0.02 | 0.88 ± 0.39 | 0.14 | 0.04 ± 0.02 | 0.05 ± 0.01 | 1.28 ± 0.73 | 0.12 |
|  | 20:4/22:5 | 856.6 |  | 0.47 ± 0.12 | 0.52 ± 0.16 | 1.11 ± 0.45 | 1.09 | 0.42 ± 0.10 | 0.53 ± 0.15 | 1.25 ± 0.47 | 1.26 |
|  | 22:1/22:6 | 888.5 |  | 0.03 ± 0.02 | 0.02 ± 0.01 | 0.64 ± 0.39 | 0.08 | 0.02 ± 0.01 | 0.02 ± 0.01 | 1.09 ± 0.61 | 0.05 |
|  | 22:5/20:3 | 858.5 |  | 0.05 ± 0.01 | 0.07 ± 0.03 | 1.38 ± 0.60 | 0.12 | 0.06 ± 0.02 | 0.06 ± 0.02 | 1.03 ± 0.43 | 0.15 |
|  | 22:5/22:6 | 880.6 |  | 0.74 ± 0.20 | 0.63 ± 0.15 | 0.85 ± 0.31 | 1.74 | 0.79 ± 0.27 | 1.36 ± 0.41 | 1.72 ± 0.78 | 3.25 |
|  | 22:6/16:1 | 804.6 |  | 0.13 ± 0.04 | 0.22 ± 0.09 | 1.73 ± 0.88 | 0.30 | 0.14 ± 0.05 | 0.12 ± 0.05 | 0.88 ± 0.45 | 0.30 |
|  | 22:6/20:3 | 856.6 |  | 0.18 ± 0.06 | 0.31 ± 0.12 | 1.72 ± 0.84 | 0.43 | 0.21 ± 0.05 | 0.17 ± 0.04 | 0.82 ± 0.26 | 0.41 |
|  | 22:6/20:4 | 854.5 | 8 | 1.90 ± 0.66 | 2.99 ± 1.11 | 1.57 ± 0.80 | 4.47 | 2.15 ± 0.54 | 2.27 ± 0.87 | 1.06 ± 0.48 | 5.45 |
|  | 22:6/22:6 | 878.5 | 9 | 2.92 ± 0.87 | 4.31 ± 1.12 | 1.48 ± 0.58 | 6.85 | 4.63 ± 1.51 | 3.77 ± 1.20 | 0.81 ± 0.37 | 9.03 |
| LPA | **14:0** | 381.4 |  | 0.00 ± 0.00 | 0.00 ± 0.00 | 0.93 ± 0.38 | 1.12 | 0.01 ± 0.00 | 0.01 ± 0.00 | **1.73 ± 0.70** | 1.30 |
| (5) | 16:0 | 409.4 | 1 | 0.15 ± 0.04 | 0.21 ± 0.07 | 1.39 ± 0.60 | 44.52 | 0.16 ± 0.05 | 0.21 ± 0.08 | 1.33 ± 0.66 | 37.16 |
|  | **18:0** | 437.5 | 2 | 0.06 ± 0.01 | 0.09 ± 0.03 | 1.60 ± 0.63 | 17.51 | 0.10 ± 0.03 | 0.14 ± 0.03 | **1.40 ± 0.48** | 23.42 |
|  | 18:1 | 435.4 | 3 | 0.06 ± 0.02 | 0.08 ± 0.02 | 1.29 ± 0.61 | 17.88 | 0.11 ± 0.03 | 0.14 ± 0.04 | 1.34 ± 0.53 | 25.73 |
|  | **18:2** | 433.4 |  | 0.06 ± 0.01 | 0.06 ± 0.02 | 0.94 ± 0.36 | 18.97 | 0.05 ± 0.01 | 0.06 ± 0.01 | **1.13 ± 0.34** | 12.40 |
| PA | 16:0/18:2 | 671.5 |  | 0.04 ± 0.01 | 0.04 ± 0.01 | 0.92 ± 0.36 | 0.30 | 0.02 ± 0.01 | 0.04 ± 0.01 | 1.57 ± 0.58 | 0.21 |
| (16) | 16:0/20:2 | 699.5 |  | 0.06 ± 0.02 | 0.04 ± 0.01 | 0.70 ± 0.22 | 0.48 | 0.04 ± 0.01 | 0.05 ± 0.01 | 1.16 ± 0.36 | 0.39 |
|  | **16:0/22:5** | 721.6 |  | 0.17 ± 0.06 | 0.31 ± 0.09 | **1.79 ± 0.78** | 1.32 | 0.48 ± 0.24 | 0.44 ± 0.14 | 0.92 ± 0.55 | 4.41 |
|  | 16:1/18:1 | 671.5 |  | 0.01 ± 0.00 | 0.01 ± 0.00 | 1.25 ± 0.58 | 0.09 | 0.01 ± 0.00 | 0.01 ± 0.00 | 0.91 ± 0.48 | 0.10 |
|  | **18:0/22:5** | 749.6 |  | 0.19 ± 0.07 | 0.30 ± 0.11 | **1.59 ± 0.78** | 1.43 | 0.17 ± 0.06 | 0.15 ± 0.03 | 0.90 ± 0.35 | 1.55 |
|  | 18:0/22:6 | 747.7 | 1 | 1.03 ± 0.25 | 1.20 ± 0.51 | 1.16 ± 0.57 | 7.78 | 0.85 ± 0.27 | 1.12 ± 0.33 | 1.33 ± 0.57 | 7.85 |
|  | 18:1/18:0 | 701.5 |  | 0.58 ± 0.16 | 0.54 ± 0.17 | 0.93 ± 0.39 | 4.41 | 0.64 ± 0.32 | 0.77 ± 0.18 | 1.20 ± 0.65 | 5.98 |
|  | 18:1/20:0 | 729.5 | 2 | 3.08 ± 0.97 | 3.22 ± 0.78 | 1.05 ± 0.42 | 23.20 | 2.78 ± 1.09 | 3.32 ± 0.68 | 1.19 ± 0.53 | 25.80 |
|  | **18:1/22:5** | 747.7 | 3 | 1.85 ± 0.56 | 3.79 ± 1.12 | **2.05 ± 0.87** | 13.98 | 1.87 ± 0.68 | 5.65 ± 2.52 | **3.02 ± 1.74** | 17.39 |
|  | 18:1/22:6 | 745.7 | 4 | 1.63 ± 0.49 | 2.25 ± 0.62 | 1.38 ± 0.56 | 12.32 | 1.20 ± 0.26 | 1.48 ± 0.36 | 1.23 ± 0.40 | 11.16 |
|  | 18:2/18:0 | 699.5 |  | 0.06 ± 0.02 | 0.08 ± 0.03 | 1.27 ± 0.63 | 0.49 | 0.08 ± 0.02 | 0.07 ± 0.02 | 0.98 ± 0.35 | 0.70 |
|  | 20:2/22:5 | 773.5 |  | 0.50 ± 0.18 | 0.35 ± 0.10 | 0.70 ± 0.33 | 3.74 | 0.36 ± 0.12 | 0.60 ± 0.20 | 1.69 ± 0.80 | 3.33 |
|  | 20:2/22:6 | 771.5 |  | 0.08 ± 0.02 | 0.09 ± 0.02 | 1.20 ± 0.44 | 0.58 | 0.08 ± 0.02 | 0.08 ± 0.02 | 1.04 ± 0.44 | 0.70 |
|  | 20:3/22:5 | 771.5 |  | 0.07 ± 0.02 | 0.05 ± 0.02 | 0.71 ± 0.38 | 0.54 | 0.05 ± 0.02 | 0.03 ± 0.00 | 0.75 ± 0.39 | 0.43 |
|  | **22:6/22:5** | 793.5 |  | 0.60 ± 0.28 | 0.49 ± 0.17 | 0.81 ± 0.47 | 4.55 | 0.40 ± 0.15 | 0.67 ± 0.14 | **1.66 ± 0.70** | 3.73 |
|  | 22:6/22:6 | 791.5 | 5 | 2.09 ± 0.85 | 1.67 ± 0.41 | 0.80 ± 0.38 | 24.79 | 1.75 ± 0.69 | 2.80 ± 0.63 | 1.60 ± 0.73 | 16.25 |
| DAG | 14:0,18:1 | 584.5 |  | 0.32 ± 0.10 | 0.56 ± 0.09 | 1.74 ± 0.60 | 2.31 | 0.18 ± 0.07 | 0.33 ± 0.06 | 1.81 ± 0.76 | 1.66 |
| (24) | 14:0,18:2 | 582.6 | 1 | 1.25 ± 0.14 | 1.35 ± 0.23 | 1.08 ± 0.22 | 8.96 | 0.68 ± 0.21 | 1.69 ± 0.44 | 2.50 ± 1.01 | 6.24 |
|  | **16:0,16:0** | 586.5 |  | 0.27 ± 0.02 | 0.29 ± 0.10 | 1.08 ± 0.38 | 1.95 | 0.12 ± 0.03 | 0.45 ± 0.04 | **3.68 ± 0.97** | 1.12 |
|  | 16:0,16:1 | 584.5 |  | 0.11 ± 0.01 | 0.14 ± 0.05 | 1.36 ± 0.46 | 0.76 | 0.08 ± 0.02 | 0.11 ± 0.02 | 1.33 ± 0.36 | 0.78 |
|  | 16:0,18:0 | 614.5 |  | 0.11 ± 0.01 | 0.12 ± 0.01 | 1.16 ± 0.19 | 0.75 | 0.10 ± 0.03 | 0.13 ± 0.02 | 1.36 ± 0.43 | 0.91 |
|  | 16:0,18:1 | 612.5 |  | 0.04 ± 0.01 | 0.06 ± 0.02 | 1.27 ± 0.43 | 0.32 | 0.03 ± 0.01 | 0.04 ± 0.01 | 1.47 ± 0.51 | 0.28 |
|  | 16:0,18:2 | 610.3 |  | 0.02 ± 0.01 | 0.02 ± 0.01 | 1.23 ± 0.44 | 0.14 | 0.01 ± 0.00 | 0.02 ± 0.00 | 1.38 ± 0.39 | 0.13 |
|  | **16:0,20:1** | 640.6 | 2 | 1.64 ± 0.25 | 1.60 ± 0.29 | 0.98 ± 0.23 | 11.70 | 1.13 ± 0.23 | 1.88 ± 0.46 | **1.66 ± 0.53** | 10.40 |
|  | 16:0,24:1 | 696.6 | 3 | 1.03 ± 0.10 | 1.19 ± 0.21 | 1.15 ± 0.23 | 7.36 | 0.95 ± 0.27 | 1.04 ± 0.20 | 1.09 ± 0.38 | 8.74 |
|  | 16:1,18:1 | 610.3 |  | 0.02 ± 0.00 | 0.02 ± 0.00 | 1.20 ± 0.36 | 0.13 | 0.01 ± 0.01 | 0.02 ± 0.00 | 1.42 ± 0.59 | 0.13 |
|  | 16:1,18:2 | 608.5 |  | 0.14 ± 0.03 | 0.16 ± 0.02 | 1.15 ± 0.25 | 0.97 | 0.13 ± 0.03 | 0.21 ± 0.08 | 1.63 ± 0.72 | 1.16 |
|  | 16:1,20:1 | 638.5 |  | 0.32 ± 0.03 | 0.39 ± 0.11 | 1.20 ± 0.35 | 2.32 | 0.22 ± 0.06 | 0.33 ± 0.05 | 1.47 ± 0.43 | 2.06 |
|  | 16:1,22:1 | 666.6 | 4 | 0.58 ± 0.21 | 0.85 ± 0.21 | 1.47 ± 0.64 | 7.30 | 0.54 ± 0.08 | 0.67 ± 0.13 | 1.24 ± 0.30 | 4.95 |
|  | 18:0,18:1 | 640.6 |  | 0.04 ± 0.01 | 0.04 ± 0.01 | 1.08 ± 0.37 | 0.28 | 0.03 ± 0.01 | 0.04 ± 0.00 | 1.24 ± 0.33 | 0.26 |
|  | 18:0,22:6 | 686.4 |  | 0.01 ± 0.00 | 0.01 ± 0.00 | 1.55 ± 0.44 | 0.05 | 0.01 ± 0.00 | 0.01 ± 0.00 | 1.22 ± 0.37 | 0.10 |
|  | 18:1,18:1 | 638.5 |  | 0.01 ± 0.00 | 0.01 ± 0.00 | 1.26 ± 0.33 | 0.08 | 0.01 ± 0.00 | 0.01 ± 0.00 | 1.68 ± 0.73 | 0.06 |
|  | 18:1,18:2 | 636.6 |  | 0.01 ± 0.00 | 0.02 ± 0.01 | 1.36 ± 0.64 | 0.10 | 0.01 ± 0.00 | 0.02 ± 0.00 | 1.84 ± 0.83 | 0.09 |
|  | 18:1,20:1 | 666.6 | 5 | 0.87 ± 0.26 | 0.85 ± 0.18 | 0.98 ± 0.36 | 6.19 | 0.76 ± 0.21 | 0.86 ± 0.28 | 1.13 ± 0.48 | 6.99 |
|  | 18:1,22:1 | 694.6 | 6 | 0.74 ± 0.17 | 1.20 ± 0.21 | 1.63 ± 0.48 | 5.26 | 0.55 ± 0.09 | 0.88 ± 0.22 | 1.59 ± 0.47 | 5.07 |
|  | 18:1,22:6 | 684.5 |  | 0.00 ± 0.00 | 0.01 ± 0.00 | 1.55 ± 0.40 | 0.02 | 0.00 ± 0.00 | 0.01 ± 0.00 | 1.22 ± 0.47 | 0.05 |
|  | **18:2,18:2** | 634.6 |  | 0.14 ± 0.04 | 0.17 ± 0.02 | 1.21 ± 0.33 | 1.02 | 0.09 ± 0.02 | 0.17 ± 0.03 | **1.96 ± 0.58** | 0.81 |
|  | **18:2,20:1** | 664.6 | 7 | 0.88 ± 0.28 | 0.93 ± 0.17 | 1.06 ± 0.39 | 6.26 | 0.47 ± 0.12 | 1.10 ± 0.10 | **2.31 ± 0.63** | 4.37 |
|  | **18:2,22:6** | 682.5 | 8 | 4.72 ± 0.50 | 5.24 ± 1.33 | 1.11 ± 0.31 | 33.75 | 4.49 ± 1.19 | 7.83 ± 1.98 | **1.74 ± 0.64** | 41.33 |
|  | 20:4,20:4 | 682.5 |  | 0.28 ± 0.11 | 0.25 ± 0.02 | 0.89 ± 0.37 | 2.03 | 0.25 ± 0.04 | 0.33 ± 0.04 | 1.30 ± 0.27 | 2.33 |
| TAG | 42:0 | 740.6 |  | 0.24 ± 0.05 | 0.27 ± 0.06 | 1.13 ± 0.36 | 0.12 | 0.21 ± 0.05 | 0.27 ± 0.05 | 1.26 ± 0.38 | 0.17 |
| (65  /121*) | **42:1** | 738.7 |  | 0.08 ± 0.01 | 0.07 ± 0.00 | 0.84 ± 0.13 | 0.04 | 0.05 ± 0.01 | 0.07 ± 0.02 | **1.44 ± 0.41** | 0.04 |
|  | 42:2 | 736.7 |  | 0.02 ± 0.00 | 0.03 ± 0.01 | 1.07 ± 0.31 | 0.01 | 0.02 ± 0.00 | 0.03 ± 0.00 | 1.13 ± 0.30 | 0.02 |
|  | **44:0** | 768.7 |  | 0.10 ± 0.03 | 0.10 ± 0.02 | 1.02 ± 0.35 | 0.05 | 0.07 ± 0.01 | 0.11 ± 0.02 | **1.71 ± 0.44** | 0.05 |
|  | 44:1 | 766.7 |  | 0.35 ± 0.11 | 0.29 ± 0.09 | 0.85 ± 0.38 | 0.17 | 0.19 ± 0.03 | 0.35 ± 0.10 | 1.79 ± 0.58 | 0.15 |
|  | **44:2** | 764.7 |  | 0.23 ± 0.08 | 0.16 ± 0.04 | 0.71 ± 0.31 | 0.11 | 0.10 ± 0.02 | 0.24 ± 0.09 | **2.41 ± 0.94** | 0.08 |
|  | 44:3 | 762.6 |  | 0.02 ± 0.00 | 0.02 ± 0.00 | 1.04 ± 0.28 | 0.01 | 0.02 ± 0.00 | 0.02 ± 0.00 | 1.30 ± 0.33 | 0.01 |
|  | **46:0** | 796.8 | 1 | 4.69 ± 2.00 | 3.65 ± 1.00 | 0.78 ± 0.39 | 2.31 | 0.94 ± 0.14 | 3.34 ± 0.77 | **3.56 ± 0.98** | 0.74 |
|  | 46:1 | 794.8 |  | 1.26 ± 0.31 | 1.34 ± 0.26 | 1.06 ± 0.33 | 0.62 | 1.02 ± 0.22 | 1.46 ± 0.28 | 1.43 ± 0.41 | 0.80 |
|  | 46:2 | 792.7 |  | 0.43 ± 0.08 | 0.47 ± 0.10 | 1.09 ± 0.31 | 0.21 | 0.36 ± 0.09 | 0.65 ± 0.17 | 1.81 ± 0.66 | 0.28 |
|  | 46:3 | 790.7 |  | 0.32 ± 0.08 | 0.18 ± 0.06 | 0.57 ± 0.23 | 0.16 | 0.13 ± 0.03 | 0.25 ± 0.08 | 1.88 ± 0.73 | 0.10 |
|  | 46:4 | 788.7 |  | 0.02 ± 0.01 | 0.02 ± 0.00 | 1.12 ± 0.36 | 0.01 | 0.02 ± 0.00 | 0.02 ± 0.00 | 1.00 ± 0.24 | 0.02 |
|  | 48:0 | 824.7 | 2 | 10.73 ± 2.20 | 10.17 ± 2.29 | 0.95 ± 0.29 | 5.28 | 6.91 ± 1.18 | 9.50 ± 1.80 | 1.37 ± 0.35 | 5.41 |
|  | 48:1 | 822.8 | 3 | 4.79 ± 1.03 | 4.97 ± 0.60 | 1.04 ± 0.26 | 2.36 | 3.22 ± 0.37 | 4.41 ± 0.68 | 1.37 ± 0.26 | 2.52 |
|  | 48:2 | 820.8 |  | 1.41 ± 0.43 | 1.42 ± 0.35 | 1.01 ± 0.39 | 0.70 | 1.11 ± 0.28 | 1.66 ± 0.26 | 1.50 ± 0.45 | 0.87 |
|  | 48:3 | 818.6 |  | 0.76 ± 0.12 | 0.81 ± 0.10 | 1.06 ± 0.22 | 0.38 | 0.60 ± 0.08 | 0.97 ± 0.16 | 1.62 ± 0.33 | 0.47 |
|  | 48:4 | 816.7 |  | 0.11 ± 0.03 | 0.09 ± 0.01 | 0.74 ± 0.22 | 0.06 | 0.07 ± 0.01 | 0.12 ± 0.04 | 1.64 ± 0.58 | 0.06 |
|  | **50:0** | 852.8 | 4 | 5.23 ± 0.91 | 5.13 ± 1.03 | 0.98 ± 0.26 | 2.57 | 2.93 ± 0.55 | 6.16 ± 0.90 | **2.10 ± 0.50** | 2.30 |
|  | **50:1** | 850.8 | 5 | 10.47 ± 2.45 | 10.33 ± 2.08 | 0.99 ± 0.31 | 5.15 | 6.25 ± 1.57 | 11.46 ± 1.88 | **1.83 ± 0.55** | 4.90 |
|  | **50:2** | 848.7 | 6 | 14.09 ± 1.99 | 15.23 ± 1.69 | 1.08 ± 0.19 | 6.94 | 10.06 ± 1.05 | 14.50 ± 1.65 | **1.44 ± 0.22** | 7.88 |
|  | 50:3 | 846.8 |  | 0.73 ± 0.14 | 0.62 ± 0.07 | 0.85 ± 0.19 | 0.36 | 0.41 ± 0.07 | 0.53 ± 0.06 | 1.29 ± 0.26 | 0.32 |
|  | 50:4 | 844.8 |  | 0.36 ± 0.11 | 0.32 ± 0.05 | 0.88 ± 0.29 | 0.18 | 0.28 ± 0.04 | 0.35 ± 0.03 | 1.25 ± 0.23 | 0.22 |
|  | 50:5 | 842.7 |  | 0.50 ± 0.18 | 0.33 ± 0.07 | 0.66 ± 0.27 | 0.25 | 0.32 ± 0.05 | 0.36 ± 0.08 | 1.15 ± 0.31 | 0.25 |
|  | 50:6 | 840.7 |  | 0.01 ± 0.00 | 0.01 ± 0.00 | 1.08 ± 0.27 | 0.01 | 0.01 ± 0.00 | 0.01 ± 0.00 | 1.16 ± 0.31 | 0.01 |
|  | 52:0 | 880.7 |  | 2.09 ± 0.60 | 1.85 ± 0.51 | 0.88 ± 0.35 | 1.03 | 1.44 ± 0.18 | 2.31 ± 0.28 | 1.60 ± 0.28 | 1.13 |
|  | **52:1** | 878.8 | 7 | 12.90 ± 2.67 | 12.25 ± 1.98 | 0.95 ± 0.25 | 6.35 | 7.90 ± 1.13 | 13.21 ± 2.13 | **1.67 ± 0.36** | 6.19 |
|  | **52:2** | 876.8 | 8 | 13.97 ± 2.41 | 14.94 ± 1.99 | 1.07 ± 0.23 | 6.88 | 8.97 ± 1.11 | 14.88 ± 1.70 | **1.66 ± 0.28** | 7.02 |
|  | 52:3 | 874.8 | 9 | 17.46 ± 3.92 | 16.82 ± 2.58 | 0.96 ± 0.26 | 8.59 | 12.84 ± 2.60 | 19.28 ± 2.64 | 1.50 ± 0.37 | 10.05 |
|  | 52:4 | 872.7 | 10 | 4.76 ± 1.46 | 3.95 ± 0.92 | 0.83 ± 0.32 | 2.34 | 3.04 ± 0.39 | 4.77 ± 0.62 | 1.57 ± 0.29 | 2.38 |
|  | 52:5 | 870.8 |  | 1.61 ± 0.28 | 1.54 ± 0.22 | 0.96 ± 0.21 | 0.79 | 0.95 ± 0.16 | 1.84 ± 0.39 | 1.94 ± 0.53 | 0.74 |
|  | 52:6 | 868.7 |  | 0.49 ± 0.11 | 0.35 ± 0.05 | 0.73 ± 0.20 | 0.24 | 0.33 ± 0.05 | 0.27 ± 0.06 | 0.81 ± 0.23 | 0.26 |
|  | 52:7 | 866.7 |  | 0.01 ± 0.00 | 0.01 ± 0.00 | 0.66 ± 0.24 | 0.01 | 0.01 ± 0.00 | 0.01 ± 0.00 | 1.16 ± 0.34 | 0.01 |
|  | **54:0** | 908.8 |  | 0.70 ± 0.07 | 0.78 ± 0.10 | 1.12 ± 0.19 | 0.34 | 0.43 ± 0.03 | 0.71 ± 0.10 | **1.65 ± 0.25** | 0.34 |
|  | **54:1** | 906.7 | 11 | 6.40 ± 0.55 | 7.37 ± 1.21 | 1.15 ± 0.21 | 3.15 | 4.07 ± 0.45 | 6.90 ± 0.58 | **1.69 ± 0.23** | 3.19 |
|  | 54:2 | 904.8 | 12 | 6.45 ± 0.48 | 6.73 ± 0.96 | 1.04 ± 0.17 | 3.17 | 4.10 ± 0.79 | 6.16 ± 0.28 | 1.50 ± 0.30 | 3.21 |
|  | **54:3** | 902.7 | 13 | 32.43 ± 7.53 | 30.79 ± 4.36 | 0.95 ± 0.26 | 15.97 | 18.49 ± 2.65 | 29.70 ± 3.25 | **1.61 ± 0.29** | 14.48 |
|  | 54:4 | 900.8 | 14 | 5.33 ± 1.41 | 5.43 ± 0.73 | 1.02 ± 0.30 | 2.63 | 3.34 ± 0.29 | 5.06 ± 0.81 | 1.52 ± 0.28 | 2.61 |
|  | **54:5** | 898.7 | 15 | 7.69 ± 1.79 | 7.34 ± 1.15 | 0.96 ± 0.27 | 3.78 | 4.05 ± 0.41 | 7.94 ± 1.59 | **1.96 ± 0.44** | 3.18 |
|  | 54:6 | 896.7 | 16 | 3.95 ± 1.05 | 3.28 ± 0.95 | 0.83 ± 0.33 | 1.94 | 1.96 ± 0.30 | 3.99 ± 1.51 | 2.03 ± 0.83 | 1.54 |
|  | 54:7 | 894.7 |  | 0.09 ± 0.03 | 0.07 ± 0.02 | 0.78 ± 0.29 | 0.05 | 0.05 ± 0.01 | 0.09 ± 0.03 | 2.05 ± 0.88 | 0.04 |
|  | 54:8 | 892.7 |  | 0.16 ± 0.04 | 0.12 ± 0.02 | 0.74 ± 0.24 | 0.08 | 0.07 ± 0.01 | 0.12 ± 0.02 | 1.79 ± 0.43 | 0.05 |
|  | **54:9** | 890.5 |  | 0.33 ± 0.06 | 0.37 ± 0.05 | 1.11 ± 0.25 | 0.16 | 0.23 ± 0.04 | 0.38 ± 0.05 | **1.68 ± 0.36** | 0.18 |
|  | 56:1 | 934.8 |  | 0.46 ± 0.08 | 0.45 ± 0.08 | 0.98 ± 0.25 | 0.23 | 0.35 ± 0.06 | 0.48 ± 0.06 | 1.38 ± 0.30 | 0.27 |
|  | 56:2 | 932.8 |  | 1.12 ± 0.20 | 1.07 ± 0.18 | 0.96 ± 0.24 | 0.55 | 0.83 ± 0.13 | 1.10 ± 0.13 | 1.32 ± 0.26 | 0.65 |
|  | 56:3 | 930.8 | 17 | 3.36 ± 0.54 | 2.57 ± 0.34 | 0.76 ± 0.16 | 1.65 | 2.27 ± 0.29 | 3.30 ± 0.65 | 1.45 ± 0.34 | 1.78 |
|  | **56:4** | 928.7 |  | 1.49 ± 0.27 | 1.49 ± 0.23 | 1.00 ± 0.24 | 0.73 | 0.76 ± 0.16 | 1.45 ± 0.24 | **1.90 ± 0.52** | 0.60 |
|  | 56:5 | 926.8 |  | 0.18 ± 0.02 | 0.18 ± 0.03 | 0.99 ± 0.22 | 0.09 | 0.14 ± 0.03 | 0.16 ± 0.03 | 1.20 ± 0.34 | 0.11 |
|  | 56:6 | 924.7 |  | 0.17 ± 0.05 | 0.16 ± 0.02 | 0.95 ± 0.27 | 0.08 | 0.10 ± 0.02 | 0.16 ± 0.02 | 1.69 ± 0.35 | 0.08 |
|  | 56:7 | 922.7 |  | 0.19 ± 0.01 | 0.16 ± 0.04 | 0.82 ± 0.23 | 0.10 | 0.07 ± 0.01 | 0.19 ± 0.07 | 2.57 ± 0.97 | 0.06 |
|  | 56:8 | 920.7 |  | 1.18 ± 0.33 | 0.86 ± 0.14 | 0.73 ± 0.24 | 0.58 | 0.53 ± 0.09 | 1.01 ± 0.11 | 1.92 ± 0.40 | 0.41 |
|  | 56:9 | 918.7 |  | 1.38 ± 0.22 | 1.32 ± 0.18 | 0.95 ± 0.20 | 0.68 | 1.02 ± 0.10 | 1.44 ± 0.24 | 1.42 ± 0.28 | 0.80 |
|  | 56:10 | 916.8 |  | 0.41 ± 0.10 | 0.43 ± 0.09 | 1.06 ± 0.34 | 0.20 | 0.25 ± 0.03 | 0.42 ± 0.06 | 1.70 ± 0.33 | 0.20 |
|  | 58:3 | 958.7 |  | 0.57 ± 0.14 | 0.48 ± 0.09 | 0.84 ± 0.26 | 0.28 | 0.40 ± 0.03 | 0.52 ± 0.08 | 1.30 ± 0.24 | 0.31 |
|  | 58:4 | 956.7 |  | 0.27 ± 0.07 | 0.22 ± 0.04 | 0.82 ± 0.26 | 0.13 | 0.19 ± 0.03 | 0.23 ± 0.04 | 1.18 ± 0.25 | 0.15 |
|  | **58:5** | 954.8 |  | 0.03 ± 0.00 | 0.03 ± 0.00 | 0.95 ± 0.19 | 0.01 | 0.01 ± 0.00 | 0.03 ± 0.00 | **2.77 ± 0.61** | 0.01 |
|  | 58:6 | 952.8 |  | 0.10 ± 0.02 | 0.11 ± 0.01 | 1.12 ± 0.21 | 0.05 | 0.07 ± 0.02 | 0.11 ± 0.02 | 1.54 ± 0.44 | 0.06 |
|  | **58:8** | 948.6 |  | 0.18 ± 0.03 | 0.18 ± 0.01 | 1.02 ± 0.20 | 0.09 | 0.10 ± 0.02 | 0.21 ± 0.03 | **2.06 ± 0.56** | 0.08 |
|  | **58:9** | 946.6 |  | 0.42 ± 0.09 | 0.36 ± 0.09 | 0.85 ± 0.27 | 0.21 | 0.21 ± 0.01 | 0.48 ± 0.10 | **2.29 ± 0.51** | 0.17 |
|  | 58:10 | 944.7 |  | 0.40 ± 0.13 | 0.28 ± 0.08 | 0.69 ± 0.29 | 0.20 | 0.15 ± 0.05 | 0.22 ± 0.04 | 1.43 ± 0.56 | 0.12 |
|  | 60:2 | 988.8 |  | 0.06 ± 0.01 | 0.07 ± 0.01 | 1.10 ± 0.23 | 0.03 | 0.04 ± 0.00 | 0.07 ± 0.01 | 1.59 ± 0.19 | 0.03 |
|  | 60:3 | 986.9 |  | 0.10 ± 0.02 | 0.09 ± 0.01 | 0.93 ± 0.21 | 0.05 | 0.06 ± 0.01 | 0.09 ± 0.01 | 1.58 ± 0.30 | 0.05 |
|  | 60:4 | 984.7 |  | 0.29 ± 0.08 | 0.24 ± 0.06 | 0.83 ± 0.30 | 0.14 | 0.20 ± 0.06 | 0.29 ± 0.07 | 1.48 ± 0.59 | 0.15 |
|  | **60:10** | 972.7 | 18 | 8.40 ± 1.12 | 9.26 ± 1.25 | 1.10 ± 0.21 | 4.14 | 6.09 ± 0.98 | 10.13 ± 0.93 | **1.66 ± 0.31** | 4.77 |
|  | **60:11** | 970.6 | 19 | 8.40 ± 0.85 | 9.24 ± 1.23 | 1.10 ± 0.18 | 4.14 | 6.15 ± 1.04 | 9.70 ± 0.81 | **1.58 ± 0.30** | 4.82 |
|  | 60:16 | 960.6 |  | 0.16 ± 0.03 | 0.21 ± 0.04 | 1.37 ± 0.36 | 0.08 | 0.13 ± 0.03 | 0.22 ± 0.05 | 1.74 ± 0.62 | 0.10 |
| ST | **d18:0/24:1** | 890.7 |  | 0.05 ± 0.01 | 0.06 ± 0.03 | 1.26 ± 0.62 | 4.78 | 0.04 ± 0.01 | 0.11 ± 0.03 | **2.71 ± 0.99** | 6.54 |
| (8) | d18:0/24:6 | 880.6 | 1 | 0.12 ± 0.05 | 0.14 ± 0.04 | 1.13 ± 0.54 | 12.58 | 0.08 ± 0.02 | 0.14 ± 0.03 | 1.88 ± 0.65 | 12.63 |
|  | **d18:1/16:0** | 778.6 | 2 | 0.20 ± 0.06 | 0.15 ± 0.05 | 0.74 ± 0.34 | 20.55 | 0.04 ± 0.01 | 0.18 ± 0.03 | **5.01 ± 1.71** | 6.00 |
|  | d18:1/18:0 | 806.6 | 3 | 0.15 ± 0.03 | 0.21 ± 0.04 | 1.47 ± 0.40 | 14.66 | 0.14 ± 0.02 | 0.24 ± 0.06 | 1.71 ± 0.49 | 23.62 |
|  | d18:1/22:0 | 862.5 | 4 | 0.15 ± 0.04 | 0.14 ± 0.04 | 0.97 ± 0.40 | 14.72 | 0.11 ± 0.03 | 0.28 ± 0.08 | 2.48 ± 0.96 | 18.87 |
|  | d18:1/22:1 | 860.7 |  | 0.10 ± 0.02 | 0.15 ± 0.04 | 1.51 ± 0.55 | 9.90 | 0.06 ± 0.02 | 0.16 ± 0.06 | 2.67 ± 1.24 | 10.20 |
|  | **d18:1/24:0** | 890.7 | 5 | 0.14 ± 0.05 | 0.18 ± 0.07 | 1.25 ± 0.65 | 14.24 | 0.09 ± 0.02 | 0.31 ± 0.09 | **3.51 ± 1.41** | 14.62 |
|  | **d20:1/20:3** | 856.5 |  | 0.09 ± 0.02 | 0.07 ± 0.02 | 0.83 ± 0.30 | 8.58 | 0.04 ± 0.01 | 0.07 ± 0.03 | **1.61 ± 0.73** | 7.52 |
| SM | d18:0/18:0 | 733.6 |  | 8.14 ± 0.66 | 10.45 ± 1.61 | 1.28 ± 0.22 | 7.52 | 5.83 ± 0.63 | 9.28 ± 1.30 | 1.59 ± 0.28 | 5.45 |
| (10) | **d18:0/23:0** | 803.6 |  | 0.69 ± 0.12 | 0.66 ± 0.10 | 0.97 ± 0.23 | 0.63 | 0.60 ± 0.12 | 1.02 ± 0.16 | **1.72 ± 0.43** | 0.56 |
|  | d18:1/18:0 | 731.7 | 1 | 11.70 ± 1.21 | 15.77 ± 1.87 | 1.35 ± 0.21 | 10.80 | 8.25 ± 1.04 | 11.78 ± 0.81 | 1.43 ± 0.21 | 7.70 |
|  | d18:1/20:0 | 759.7 | 2 | 20.80 ± 2.29 | 27.03 ± 4.53 | 1.30 ± 0.26 | 19.21 | 15.83 ± 2.48 | 28.46 ± 1.91 | 1.80 ± 0.31 | 14.78 |
|  | d18:1/22:0 | 787.6 | 3 | 10.92 ± 3.26 | 11.44 ± 1.51 | 1.05 ± 0.34 | 10.09 | 16.99 ± 2.45 | 16.76 ± 2.49 | 0.99 ± 0.20 | 15.87 |
|  | d18:1/22:1 | 785.6 | 4 | 11.01 ± 1.11 | 14.28 ± 1.66 | 1.30 ± 0.20 | 10.17 | 9.09 ± 1.05 | 16.69 ± 1.18 | 1.84 ± 0.25 | 8.49 |
|  | d18:1/23:0 | 801.7 |  | 4.41 ± 0.89 | 3.44 ± 0.50 | 0.78 ± 0.19 | 4.08 | 4.41 ± 1.31 | 6.75 ± 1.22 | 1.53 ± 0.53 | 4.11 |
|  | d18:1/24:0 | 815.6 | 5 | 12.37 ± 2.94 | 12.21 ± 1.74 | 0.99 ± 0.27 | 11.43 | 19.45 ± 4.73 | 22.23 ± 4.67 | 1.14 ± 0.37 | 18.16 |
|  | d18:1/24:1 | 813.6 | 6 | 13.17 ± 2.09 | 16.07 ± 2.39 | 1.22 ± 0.27 | 12.17 | 13.94 ± 2.60 | 21.14 ± 3.13 | 1.52 ± 0.36 | 13.01 |
|  | d18:1/24:2 | 811.6 | 7 | 15.05 ± 4.27 | 17.77 ± 1.70 | 1.18 ± 0.35 | 13.90 | 12.71 ± 3.57 | 21.6 ± 2.98 | 1.70 ± 0.53 | 11.87 |
| Cer | d18:0/18:0 | 568.7 |  | 0.27 ± 0.10 | 0.95 ± 0.12 | 3.48 ± 1.35 | 1.07 | 0.39 ± 0.21 | 0.40 ± 0.06 | 1.03 ± 0.57 | 1.25 |
| (9) | **d18:0/22:0** | 624.7 |  | 0.24 ± 0.05 | 0.43 ± 0.11 | **1.82 ± 0.62** | 0.92 | 0.22 ± 0.09 | 0.29 ± 0.08 | 1.28 ± 0.61 | 0.72 |
|  | **d18:0/24:0** | 652.5 |  | 0.93 ± 0.29 | 1.96 ± 0.30 | **2.11 ± 0.74** | 3.60 | 0.88 ± 0.25 | 1.39 ± 0.52 | 1.58 ± 0.74 | 2.83 |
|  | d18:1/18:0 | 566.7 | 1 | 9.06 ± 3.22 | 40.39 ± 2.37 | 4.46 ± 1.61 | 35.28 | 13.55 ± 4.31 | 15.04 ± 2.93 | 1.11 ± 0.41 | 43.75 |
|  | d18:1/20:5 | 580.7 |  | 0.35 ± 0.04 | 1.19 ± 0.66 | 3.43 ± 1.95 | 1.35 | 0.36 ± 0.12 | 0.28 ± 0.04 | 0.79 ± 0.29 | 1.15 |
|  | d18:1/22:0 | 622.7 | 2 | 2.93 ± 1.04 | 4.90 ± 1.31 | 1.67 ± 0.74 | 11.41 | 3.68 ± 1.06 | 5.36 ± 1.08 | 1.45 ± 0.51 | 11.90 |
|  | d18:1/24:0 | 650.7 | 3 | 3.32 ± 0.85 | 6.14 ± 1.59 | 1.85 ± 0.67 | 12.94 | 5.70 ± 1.14 | 7.68 ± 2.42 | 1.35 ± 0.50 | 18.39 |
|  | d18:1/24:1 | 648.7 | 4 | 7.87 ± 1.56 | 13.57 ± 4.69 | 1.72 ± 0.69 | 30.66 | 5.10 ± 2.12 | 8.59 ± 2.09 | 1.69 ± 0.81 | 16.46 |
|  | d18:1/24:2 | 646.7 |  | 0.71 ± 0.23 | 1.30 ± 0.28 | 1.82 ± 0.71 | 2.77 | 1.10 ± 0.33 | 1.26 ± 0.33 | 1.14 ± 0.45 | 3.55 |
| MHC | d18:0/20:0 | 758.7 |  | 0.59 ± 0.22 | 0.63 ± 0.22 | 1.07 ± 0.54 | 3.38 | 1.35 ± 0.43 | 1.47 ± 0.19 | 1.09 ± 0.37 | 6.54 |
| (7) | **d18:0/24:0** | 814.7 |  | 0.18 ± 0.05 | 0.50 ± 0.07 | **2.74 ± 0.79** | 1.05 | 0.18 ± 0.05 | 0.26 ± 0.10 | 1.47 ± 0.68 | 0.87 |
|  | d18:1/20:0 | 756.7 |  | 0.54 ± 0.12 | 0.91 ± 0.28 | 1.69 ± 0.64 | 3.07 | 0.70 ± 0.19 | 0.70 ± 0.14 | 1.00 ± 0.34 | 3.41 |
|  | **d18:1/22:0** | 784.7 | 1 | 1.69 ± 0.54 | 6.97 ± 0.85 | **4.12 ± 1.41** | 9.68 | 3.08 ± 0.90 | 3.41 ± 1.22 | 1.11 ± 0.51 | 14.92 |
|  | **d18:1/24:0** | 812.7 | 2 | 5.33 ± 1.43 | 16.21 ± 2.89 | **3.04 ± 0.98** | 30.51 | 7.03 ± 2.05 | 9.47 ± 2.90 | 1.35 ± 0.57 | 34.07 |
|  | d18:1/24:1 | 810.6 | 3 | 8.24 ± 1.55 | 9.90 ± 4.38 | 1.20 ± 0.58 | 47.16 | 7.48 ± 2.61 | 4.06 ± 1.22 | 0.54 ± 0.25 | 36.24 |
|  | d18:1/24:2 | 808.6 |  | 0.90 ± 0.35 | 1.76 ± 0.32 | 1.96 ± 0.85 | 5.14 | 0.82 ± 0.25 | 2.01 ± 0.45 | 2.47 ± 0.95 | 3.96 |

Table S3. Isomeric acyl chains of a) PC, b) PE and c) TAG species. Acyl chain locations of PC and PE were confirmed by CID spectra. For TG, exact location of each acyl chain in a molecule is not differentiated.

a) PC

| Molecular species | possible acyl chains | m/z |  | Molecular species | possible acyl chains | m/z |  | Molecular species | possible acyl chains | m/z |
| --- | --- | --- | --- | --- | --- | --- | --- | --- | --- | --- |
| 30:0 | 16:0/14:0 | 706.5 |  | 34:4 | 14:0/20:4 | 754.5 |  | 38:5 | 16:0/22:5 | 808.5 |
| 30:1 | 16:1/14:0 | 704.5 |  | 36:1 | 18:0/18:1 | 788.5 |  |  | 18:1/20:4 |  |
| 32:0 | 16:0/16:0 | 734.6 |  | 36:2 | 16:0/20:2 | 786.6 |  | 38:6 | 16:1/22:5 | 806.5 |
| 32:1 | 16:1/16:0 | 732.7 |  |  | 18:1/18:1 |  |  |  | 16:0/22:6 |  |
|  | 16:0/16:1 |  |  |  | 18:0/18:2 |  |  |  | 18:2/20:4 |  |
| 32:2 | 14:0/18:2 | 730.6 |  | 36:3 | 16:0/20:3 | 784.6 |  | 38:7 | 16:1/22:6 | 804.5 |
|  | 16:1/16:1 |  |  |  | 18:1/18:2 |  |  | 40:5 | 18:0/22:5 | 836.5 |
|  | 18:2/14:0 |  |  | 36:4 | 16:0/20:4 | 782.5 |  | 40:6 | 16:0/24:6 | 834.5 |
| 34:0 | 18:0/16:0 | 762.6 |  |  | 18:2/18:2 |  |  |  | 18:1/22:5 |  |
| 34:1 | 16:0/18:1 | 760.4 |  | 36:5 | 16:1/20:4 | 780.5 |  |  | 18:0/22:6 |  |
|  | 18:1/16:0 |  |  |  | 16:0/20:5 |  |  | 40:7 | 18:1/22:6 | 832.6 |
| 34:2 | 16:1/18:1 | 758.6 |  | 36:6 | 14:0/22:6 | 778.6 |  |  | 18:2/22:5 |  |
|  | 16:0/18:2 |  |  | 38:3 | 18:0/20:3 | 812.6 |  | 40:8 | 18:2/22:6 | 830.5 |
| 34:3 | 16:1/18:2 | 756.5 |  | 38:4 | 16:0/22:4 | 810.5 |  | 42:10 | 22:6/20:4 | 854.5 |
|  | 16:0/18:3 |  |  |  | 18:1/20:3 |  |  |  |  |  |
|  | 18:3/16:0 |  |  |  | 18:0/20:4 |  |  |  |  |  |

b) PE

| Molecular species | possible acyl chains | m/z |  | Molecular species | possible acyl chains | m/z |  | Molecular species | possible acyl chains | m/z |
| --- | --- | --- | --- | --- | --- | --- | --- | --- | --- | --- |
| 34:0 | 18:0/16:0 | 720.6 |  | 36:7 | 16:1/20:6 | 734.6 |  | 40:7 | 18:1/22:6 | 790.5 |
| 34:1 | 18:1/16:0 | 718.5 |  | 38:4 | 20:4/18:0 | 768.5 |  | 40:8 | 22:6/18:2 | 788.5 |
| 36:1 | 16:0/20:1 | 746.6 |  | 38:5 | 18:1/20:4 | 766.6 |  |  | 18:2/22:6 |  |
|  | 20:1/16:0 |  |  | 38:6 | 16:0/22:6 | 764.5 |  | 40:12 | 20:6/20:6 | 780.4 |
| 36:2 | 18:1/18:1 | 744.5 |  |  | 22:6/16:0 |  |  | 42:12 | 20:6/22:6 | 808.5 |
|  | 18:2/18:0 |  |  | 38:7 | 16:1/22:6 | 762.4 |  | 44:10 | 22:6/22:4 | 840.6 |
| 36:3 | 18:1/18:2 | 742.6 |  | 40:5 | 18:0/22:5 | 794.5 |  | 44:11 | 22:6/22:5 | 838.5 |
| 36:4 | 16:0/20:4 | 740.6 |  |  | 20:1/20:4 |  |  |  |  |  |
| 36:6 | 20:6/16:0 | 736.6 |  | 40:6 | 22:6/18:0 | 792.6 |  |  |  |  |
|  | 16:0/20:6 |  |  |  | 18:0/22:6 |  |  |  |  |  |

c) TAG

| Molecular species | possible acyl chains | m/z |  | Molecular species | possible acyl chains | m/z |  | Molecular species | possible acyl chains | m/z |
| --- | --- | --- | --- | --- | --- | --- | --- | --- | --- | --- |
| 42:0 | 12:0,14:0,16:0 | 740.6 |  |  | 16:0,16:1,18:1 |  |  | 56:1 | 16:0,18:1,22:0 | 934.8 |
|  | 14:0,14:0,14:0 |  |  | 50:3 | 14:0,18:1,18:2 | 846.8 |  |  | 18:0,18:0,20:1 |  |
| 42:1 | 12:0,14:0,16:1 | 738.7 |  |  | 16:0,16:1,18:2 |  |  |  | 18:0,18:1,20:0 |  |
|  | 12:0,14:1,16:0 |  |  |  | 16:1,16:1,18:1 |  |  | 56:2 | 16:0,18:1,22:1 | 932.8 |
|  | 14:0,14:0,14:1 |  |  | 50:4 | 14:0,18:2,18:2 | 844.8 |  |  | 18:0,18:1,20:1 |  |
| 42:2 | 14:0,14:1,14:1 | 736.7 |  |  | 16:1,16:1,18:2 |  |  |  | 18:1,18:1,20:0 |  |
| 44:0 | 12:0,16:0,16:0 | 768.7 |  | 50:5 | 14:1,18:2,18:2 | 842.7 |  | 56:3 | 18:1,18:1,20:1 | 930.8 |
|  | 14:0,14:0,16:0 |  |  |  | 16:1,16:2,18:2 |  |  |  | 18:1,18:2,20:0 |  |
| 44:1 | 12:0,14:0,18:1 | 766.7 |  | 50:6 | 14:1,16:1,20:4 | 840.7 |  | 56:4 | 18:1,18:1,20:2 | 928.7 |
|  | 12:0,16:0,16:1 |  |  |  | 14:1,18:2,18:3 |  |  |  | 18:1,18:2,20:1 |  |
|  | 14:0,14:0,16:1 |  |  | 52:0 | 16:0,18:0,18:0 | 880.7 |  | 56:5 | 18:0,18:1,20:4 | 926.8 |
| 44:2 | 12:0,14:1,18:1 | 764.7 |  | 52:1 | 16:0,18:0,18:1 | 878.8 |  |  | 18:2,18:2,20:1 |  |
|  | 14:0,14:1,16:1 |  |  | 52:2 | 16:0,18:1,18:1 | 876.8 |  | 56:6 | 18:1,18:1,20:4 | 924.7 |
|  | 14:1,14:1,16:0 |  |  | 52:3 | 16:0,18:1,18:2 | 874.8 |  |  | 18:1,18:2,20:3 |  |
| 44:3 | 12:0,14:1,18:2 | 762.6 |  |  | 16:1,18:1,18:1 |  |  | 56:7 | 16:0,18:2,22:5 | 922.7 |
|  | 14:1,14:1,16:1 |  |  | 52:4 | 16:0,18:2,18:2 | 872.7 |  |  | 18:1,18:2,20:4 |  |
| 46:0 | 12:0,16:0,18:0 | 796.8 |  |  | 16:1,18:1,18:2 |  |  | 56:8 | 16:1,18:1,22:6 | 920.7 |
|  | 14:0,16:0,16:0 |  |  | 52:5 | 16:0,16:1,20:4 | 870.8 |  |  | 18:1,18:2,20:5 |  |
| 46:1 | 14:0,14:0,18:1 | 794.8 |  |  | 16:1,18:2,18:2 |  |  | 56:9 | 18:1,18:2,22:6 | 918.7 |
|  | 14:0,16:0,16:1 |  |  | 52:6 | 16:1,16:1,20:4 | 868.7 |  | 56:10 | 16:2,18:2,22:6 | 916.8 |
| 46:2 | 12:0,16:1,18:1 | 792.7 |  |  | 16:1,18:2,18:3 |  |  | 58:3 | 16:0,18:2,24:1 | 958.7 |
|  | 14:0,16:1,16:1 |  |  | 52:7 | 14:0,16:1,22:6 | 866.7 |  |  | 18:1,18:1,22:1 |  |
|  | 14:1,16:0,16:1 |  |  |  | 16:1,16:1,20:5 |  |  |  | 18:1,20:1,20:1 |  |
| 46:3 | 12:0,16:1,18:2 | 790.7 |  | 54:0 | 16:0,18:0,20:0 | 908.8 |  | 58:4 | 18:1,18:2,22:1 | 956.7 |
|  | 14:1,16:1,16:1 |  |  |  | 18:0,18:0,18:0 |  |  |  | 18:2,20:1,20:1 |  |
| 46:4 | 14:1,14:1,18:2 | 788.7 |  | 54:1 | 16:0,18:0,20:1 | 906.7 |  | 58:5 | 18:2,18:2,22:1 | 954.8 |
|  | 14:1,16:1,16:2 |  |  |  | 18:0,18:0,18:1 |  |  | 58:6 | 18:1,18:1,22:4 | 952.8 |
| 48:0 | 14:0,16:0,18:0 | 824.7 |  | 54:2 | 16:0,18:1,20:1 | 904.8 |  | 58:8 | 18:1,18:1,22:6 | 948.6 |
|  | 16:0,16:0,16:0 |  |  |  | 18:0,18:1,18:1 |  |  |  | 18:1,18:2,22:5 |  |
| 48:1 | 14:0,16:0,18:1 | 822.8 |  | 54:3 | 18:1,18:1,18:1 | 902.7 |  | 58:9 | 18:1,18:2,22:6 | 946.6 |
|  | 14:0,16:1,18:0 |  |  | 54:4 | 18:1,18:1,18:2 | 900.8 |  |  | 18:2,18:2,22:5 |  |
|  | 16:0,16:0,16:1 |  |  | 54:5 | 16:0,18:1,20:4 | 898.7 |  | 58:10 | 18:2,18:2,22:6 | 944.7 |
| 48:2 | 14:0,16:1,18:1 | 820.8 |  |  | 18:1,18:2,18:2 |  |  | 60:2 | 18:1,18:1,24:1 | 988.8 |
|  | 16:0,16:1,16:1 |  |  | 54:6 | 16:1,18:1,20:4 | 896.7 |  |  | 18:1,20:1,22:0 |  |
| 48:3 | 14:1,16:1,18:1 | 818.6 |  |  | 18:2,18:2,18:2 |  |  | 60:3 | 18:1,20:1,22:1 | 986.9 |
|  | 16:1,16:1,16:1 |  |  | 54:7 | 16:0,16:1,22:6 | 894.7 |  | 60:4 | 18:2,20:1,22:1 | 984.7 |
| 48:4 | 14:1,16:1,18:2 | 816.7 |  |  | 16:0,18:2,20:5 |  |  | 60:10 | 20:3,20:3,20:4 | 972.7 |
| 50:0 | 16:0,16:0,18:0 | 852.8 |  |  | 18:2,18:2,18:3 |  |  | 60:11 | 20:3,20:4,20:4 | 970.6 |
| 50:1 | 16:0,16:0,18:1 | 850.8 |  | 54:8 | 16:1,16:1,22:6 | 892.7 |  | 60:16 | 20:4,20:6,20:6 | 960.6 |
| 50:2 | 14:0,18:0,18:2 | 848.7 |  |  | 16:1,18:2,20:5 |  |  |  |  |  |
|  | 16:0,16:0,18:2 |  |  | 54:9 | 16:1,18:2,20:6 | 890.5 |  |  |  |  |

Table S4. List of internal standards and the precursor ion type utilized in targeted quantification.

| Class | Molecular Species | precursor ion | m/z |
| --- | --- | --- | --- |
| LPC | 17:0 | [M+H]^+^ | 510.5 |
| PC | 13:0/13:0 | [M+H]^+^ | 650.5 |
| LPE | 17:1 | [M+H]^+^ | 466.5 |
| PE | 17:0/17:0 | [M+H]^+^ | 720.5 |
| LPG | 17:1 | [M-H]- | 495.5 |
| PG | 15:0/15:0 | [M-H]^-^ | 693.5 |
| PI | 17:0/20:4 | [M-H]^-^ | 871.5 |
| LPS | 17:1 | [M-H]^-^ | 508.5 |
| PS | 17:0/20:4 | [M-H]^-^ | 796.5 |
| LPA | 17:0 | [M-H]^-^ | 421.5 |
| PA | 17:0/17:0 | [M-H]^-^ | 675.5 |
| SM | d18:1/17:0 | [M+H]^+^ | 717.5 |
| Cer | d18:1/17:0 | [M+H]^+^ | 552.5 |
| MHC(glu) | d18:1/17:0 | [M+H]^+^ | 714.5 |
| MHC(gal) | d18:1/17:0 | [M+H]^+^ | 714.5 |
| ST | d18:1/17:0 | [M+H]^+^ | 794.5 |
| DAG | 17:0/17:0 | [M+NH_4_]^+^ | 614.5 |
| TAG | 17:0/17:1/17:0 D_5_ | [M+NH_4_]^+^ | 870.7 |
| CL | (14:0)_4_ | [M-2H]^2-^ | 619.8 |

Table S5. Detailed information on mice used in this study.

| Category | gastrocnemius | | soleus | |
| --- | --- | --- | --- | --- |
|  | WT | KO | WT | KO |
| Gender | Male | Male | Male | Male |
| Food intake | Normal* | Normal* | Normal* | Normal* |
| Weight (mg) | 62.82 ± 15.94 | 61.01 ± 22.04 | 4.81 ± 0.77 | 3.96 ± 0.52 |

* Normal diet, NIH-31 from Zeigler Bros, Inc. (Gardners, PA, USA) *ad libitum* with tap water and euthanized with CO_2_ following the ‘Guide for Animal Experiments’ edited by Korean Academy of Medical Sciences and was approved by the Institutional Animal Care and Use Committee (IACUC) of Seoul National University.
